# Taxonomy and Ecology of Novel *Pythium sensu stricto* Clade B Species Isolated from Cucurbits

**DOI:** 10.3390/jof12100748

**Published:** 2026-10-05

**Authors:** Fatemeh Salmaninezhad, Pouria Sekandarpour, Sadaf Fouladfard, Santa Olga Cacciola, Reza Mostowfizadeh-Ghalamfarsa

**Affiliations:** 1 Department of Plant Protection, School of Agriculture, Shiraz University, Shiraz 7144113131, Iran; f.Salmaninezhad@shirazu.ac.ir (F.S.); pouria@hafez.shirazu.ac.ir (P.S.); sadaf.fouladfard@hafez.shirazu.ac.ir (S.F.); 2 Department of Agriculture, Food and Environment (Di3A), University of Catania, 95123 Catania, Italy

**Keywords:** *Oomycota*, pathogenicity, phylogeny, *Pythium*, taxonomy

## Abstract

*Pythium* species are globally distributed oomycetes of major ecological and phytopathological importance, yet species boundaries and character evolution within *Pythium sensu stricto* remain incompletely understood. Using an integrative taxonomic approach, we investigated oomycete diversity associated with greenhouse-grown cucurbits by combining morphology, multilocus phylogenetics, ecological analyses, and pathogenicity assays. A total of 351 oomycete isolates were recovered from root tissues, rhizosphere soil, and irrigation water, of which 196 belonged to *Pythium sensu stricto*. Multilocus analyses based on nuclear (*βtub*) and mitochondrial (*cox1*, *cox2*) loci resolved four strongly supported and genetically distinct lineages within Clade B, designated *P. macrosporangium* sp. nov., *P. melonis* sp. nov., *P. tardioosporum* sp. nov., and *P. tumidoantherum* sp. nov. Each lineage exhibited stable phylogenetic placement and diagnostic morphological traits. Notably, *P. macrosporangium* produced globose to subglobose sporangia, a feature exceptional within *Pythium sensu stricto*, in which sporangia are characteristically filamentous. Ecological analyses revealed pronounced substrate-dependent structuring, with irrigation water harboring minimal diversity and root tissues supporting the highest alpha diversity. *Pythium macrosporangium* dominated in all hosts, locations, and ecological substrates. Pathogenicity assays demonstrated that all lineages were pathogenic to cucurbit hosts, causing severe root rot and damping-off. These results reveal previously unrecognized diversity within *Pythium* Clade B and reveal a morphologically and ecologically distinctive lineage that challenges traditional diagnostic concepts of *Pythium sensu stricto*.

## 1. Introduction

Oomycetes are fungus-like organisms belonging to the phylum *Oomycota* within the kingdom *Stramenopila*, which, despite their morphological resemblance to true fungi, are phylogenetically more closely related to brown algae [1]. The most important plant-pathogenic genera within this group include *Globisporangium*, *Phytophthora*, *Phytopythium*, and *Pythium*. Under warm and humid greenhouse conditions, these pathogens cause destructive diseases, such as root and crown rot, damping-off, and fruit blight on greenhouse crops [2,3,4].

The genus *Pythium* was originally subdivided into eleven clades (A–K) based on sporangial morphology and internal transcribed spacer (ITS) sequence data [5]. Subsequent phylogenetic studies, however, demonstrated that *Pythium* is not monophyletic. As a result, the genus was reclassified into five distinct genera, including *Elongisporangium*, *Globisporangium*, *Pilasporangium*, *Phytopythium*, and *Pythium sensu stricto*, based on combined analyses of *cox2* and ITS sequences together with sporangial morphological characteristics [6]. More recent molecular investigations employing whole-genome sequencing largely corroborated this taxonomic framework, with the exception that *Pilasporangium* was subsumed into *Pythium sensu stricto* [7]. Species currently assigned to *Pythium sensu stricto* are characterized by filamentous to inflated sporangia and are further classified into four principal clades (A–D) [5]. Among the members of *Pythium sensu stricto*, Clade B represents the largest assemblage, encompassing species characterized by the production of filamentous to inflated sporangia and smooth-walled oogonia [8].

The species assigned to the Clade B of *Pythium sensu stricto*, including *P. catenulatum* V. D. Mathews, *P. dissotocum* Drechsler, *P. kashmirense* B. Paul, *P. myriotylum* Drechsler, *P. rhizo-oryzae* B. Paul, and *P. torulosum* Coker & P. Patt. have been reported as causal agents of pre- and post-emergence damping of greenhouse crops worldwide [9,10,11]. The threat posed by such pathogens is likely to intensify under future conditions. Simultaneously, climate change and the associated increases in temperature and humidity have amplified the risk of emergence of novel oomycete pathogens with broader host ranges and enhanced aggressiveness [12,13,14]. Although protected cultivation can buffer crops from external climatic fluctuations, greenhouse microclimates are still shaped by ambient conditions and management practices, while their warm and humid environments may favor the survival, spread, and establishment of oomycete pathogens.

Greenhouse vegetable production plays a pivotal role in ensuring food security and sustainable agriculture in Iran, with Fars Province serving as a major center of cucurbit cultivation under protected systems driven by climate and water constraints [15]. Despite this importance, limited attention has been given to the study of emerging oomycete pathogens affecting these crops in the region. The objective of this study is to formally describe four novel *Pythium* species belonging to Clade B that are associated with crown and root rot of greenhouse-grown cucurbits in Fars Province, Iran. This study integrates detailed morphological characterization with multilocus phylogenetic and ecological analyses to resolve their taxonomic placement and distribution. In addition, the pathogenicity and host range of these newly described species on cucurbit hosts are evaluated to assess their biological significance and potential impact on greenhouse production systems.

## 2. Materials and Methods

### 2.1. Sampling and Isolation

Cucurbit crops cultivated in greenhouse production systems across Fars Province, Iran, with particular emphasis on the Shiraz County, were surveyed through weekly sampling in multiple commercial greenhouses producing cucumber (*Cucumis sativus* L.), cantaloupe (*C. melo* L. var. *cantalupensis* Naudin), muskmelon (*C. melo* var. *reticulatus* Naudin), squash (*Cucurbita pepo* L.), and watermelon (*Citrullus lanatus* (Thunb.) Mansfeld). Sampling focused on plants exhibiting symptoms of root and crown rot, wilting, or general decline to maximize the recovery of pathogenic *Pythium* and *Pythium*-like species. From each symptomatic plant, root and crown tissues, rhizosphere soil, infected surrounding plant debris, and irrigation water were collected to capture both plant-associated and environmental inoculum. Specifically, water samples were collected using sterile 50 mL Falcon tubes. For each sampling site, a total volume of 200 mL of irrigation water was collected (comprising four 50 mL tubes per site). To reduce spatial bias and enhance species detection, samples were obtained from multiple greenhouses and from different locations within each greenhouse. This strategy allowed for a comprehensive assessment of oomycete diversity, distribution, and prevalence in the region.

Symptomatic plant tissues were first meticulously washed under a stream of tap water to remove adhering soil particles and then surface-dried between layers of sterile absorbent paper. The samples were subsequently placed onto a selective isolation medium, CMA-PARP (CMA = Corn Meal Agar; 40 g/L cornmeal extract; 15 g/L agar, Merk, Darmstadt, Germany), amended with a combination of antimicrobial agents (pimaricin at 10 µg/mL, Merk, Darmstadt, Germany, ampicillin at 200 µg/mL, Merk, Darmstadt, Germany, rifampicin at 10 µg/mL, Merk, Darmstadt, Germany, and pentachloronitrobenze (PCNB), Merk, Darmstadt, Germany; at 25 µg/mL) to inhibit the growth of fungi and bacteria [16].

For the isolation of pathogens from soil, a baiting protocol was employed. For each soil sample, an aliquot of 100 g of soil was placed into sterile 1 L plastic containers and flooded with sterile distilled water to approximately 1 cm above the soil surface. Leaf baits with a diameter of 5 mm were prepared using a punch from leaves of sour orange (*Citrus aurantium* L.), pistachio (*Pistacia vera* L.), ivy (*Hedera helix* L.), cucumber, squash, muskmelon, cantaloupe, and watermelon, and approximately 30 bait disks of the whole set of plant species were added to each container [17,18,19]. Annual bluegrass leaves (*Poa annua* L.) were also used as bait, cut into 5 mm segments, immersed in sterile distilled water, boiled for 10 min, cooled, and then approximately 30 pieces were added per sample [20]. These baits were floated on the water, and the setups were incubated at 25 °C for 40 h. After incubation, the baits were retrieved every 4 h, gently blotted dry, and transferred onto the CMA-PARP medium. The same baiting procedure and plant materials were used to recover oomycetes from irrigation water with the sole difference that 100 mL of irrigation water were added to the container instead of 100 gr of soil. During incubation, baits were removed at 4 h intervals, gently blotted dry, and transferred to CMA-PARP plates. All cultures, whether from direct plating or baiting, were routinely inspected for the emergence of mycelia. The leading edges of any growing colony was subcultured onto water agar (WA; 15 g/L agar, Merk, Darmstadt, Germany) to obtain pure isolates. Axenic cultures were then maintained on cornmeal agar slants at 15 °C for use in subsequent experimental procedures.

### 2.2. Morphology and Growth Characteristics of the Obtained Strains

The induction and observation of asexual reproductive structures, including sporangia and zoospores, were conducted using a standardized protocol. Isolates were initially grown on CMA plates that had been overlaid with one of several sterilized organic substrates: leaves of annual bluegrass, pistachio leaves, or hemp (*Cannabis sativa* L.) seeds [21]. Following an incubation period of 48 to 72 h to allow for mycelial colonization, substrate pieces were aseptically transferred into Petri dishes filled with one of three different liquid induction solutions: sterile distilled water, sterile soil extract, or distilled water supplemented with 1 g/L calcium nitrate (Ca(NO_3_)_2_, Merk, Darmstadt, Germany), as adapted from established methods [22,23]. To stimulate sporulation, the colonized substrates within the dishes were subjected to a series of three or four rinses with chilled water (4 °C), performed at 30 min intervals [23]. The entire setup was then placed at 14 °C in the dark. The formation and maturation of sporangia and the subsequent release of zoospores were assessed at 8 h intervals using light microscopy (Carl Zeiss, Oberkochen, Germany).

For the examination of sexual structures, specifically oogonia, antheridia, and oospores, a separate cultivation strategy was employed. Isolates were inoculated onto two distinct culture media: hemp seed agar (HSA; 60 g/L boiled hemp seed extract, 15 g/L agar, Merk, Darmstadt, Germany) and carrot agar (CA; 120 g/L boiled carrot extract, 15 g/L agar, Merk, Darmstadt, Germany), prepared according to the specifications of Jeffers and Martin [16]. All cultures were maintained in darkness at a temperature range of 20–23 °C. The development of oospores was tracked meticulously over an extended period of four months, with observations recorded every five days using a light microscope (Carl Zeiss, Oberkochen, Germany). For each isolate, 50 measurements of sexual and asexual structures were obtained, and their average was calculated.

Colony morphology and radial growth patterns were evaluated on five solid media: CMA, HSA, potato dextrose agar (PDA; 300 g/L potato extract, 20 g/L dextrose, 15 g/L agar, Merk, Darmstadt, Germany), malt extract agar (MEA; 40 g/L malt extract, 15 g/L agar, Merk, Darmstadt, Germany), and CA. Plates were incubated at 23 °C, and the linear growth rate was calculated once the mycelial front had advanced to cover a distance equivalent to at least half the radius of an 80 mm diameter Petri dish. To establish the cardinal temperatures for mycelial growth (minimum, optimum, and maximum), isolates were cultured on potato-carrot agar (PCA; extract of 300 g/L boiled potato, extract of 250 g/L boiled carrot, 15 g/L agar, Merk, Darmstadt, Germany) and incubated across a thermal gradient from 5 °C to 40 °C at 5-degree intervals, with an additional assessment at 37 °C. The experimental design incorporated three replicate plates for each isolate–temperature combination.

Mycelial growth data were analyzed using R statistical software (R version 4.5.2; R Core Team). All statistical procedures and graphical outputs were generated within the R environment. Data manipulation and reshaping were conducted using the packages dplyr and tidyr in tidyverse package (version 2.0.0) [24]. Statistical modeling and post hoc analyses were carried out using the packages stats (version 4.5.2; R Core Team), car (version 3.1.3) [25], emmeans (version 1.11.2) [26], and multcompView (version 0.1.10) [27]. Graphical visualization was produced using the ggplot2 package (version 3.5.2) [28]. Prior to analysis, the distribution of the raw growth data was assessed for normality using the Shapiro–Wilk test [29]. Because the raw data deviated significantly from normality, growth values were square-root-transformed to better meet the assumptions of parametric analysis [30]. A two-way analysis of variance (ANOVA) was performed on the transformed data with treatment, temperature, and their interaction included as fixed effects. Homogeneity of variances was evaluated using Levene’s test on the transformed data. Despite minor residual deviations from normality, the ANOVA was considered robust due to the perfectly balanced design and equal replication across all factor levels. Given the presence of a highly significant species × temperature interaction, post hoc comparisons among treatments were conducted separately within each temperature level. Estimated marginal means were computed using the emmeans package [26], and pairwise comparisons were adjusted using Tukey’s honestly significant difference (HSD) procedure. Compact letter displays were generated using multcompView [27] to summarize statistically homogeneous groups. For graphical presentation, transformed means and standard errors were back-transformed to the original scale prior to plotting, and plotting was conducted using the ggplot2 package [28].

### 2.3. DNA Extraction, Amplification, and Phylogeny

Genomic DNA was extracted following the protocol described by Mirsoleimani and Mostowfizadeh-Ghalamfarsa [31]. Fungal isolates were cultured in potato extract broth (PB; extract of 300 g/L boiled potato). Mycelial biomass was subsequently collected and freeze-dried prior to DNA isolation. Genomic DNA was extracted using the DNG™-PLUS DNA extraction kit (CinnaGen, Tehran, Iran) in accordance with the manufacturer’s guidelines.

For phylogenetic reconstruction, three loci were amplified: the beta tubulin gene (*βtub*) and the mitochondrial cytochrome c oxidase subunits 1 and 2 (*cox1* and *cox2*). Polymerase chain reaction (PCR) amplifications were performed using Amplicon master mix (Amplicon, Odense, Denmark), with primer information and thermal cycling parameters detailed in Supplementary Table S1. Amplification success was verified by electrophoresis of PCR products on 1% agarose gels. Purified amplicons were sequenced commercially by Codon Genetic Group (Tehran, Iran). All generated sequences were deposited in the GenBank database, and the corresponding accession numbers were obtained. Sequence chromatograms were assembled, edited, and curated in Geneious Prime 2022 [32]. Individual locus alignments (*βtub*, *cox1*, and *cox2*) were generated using MAFFT v.7 [33] and concatenated into a multi-locus dataset. Congruence among locus topologies was verified before concatenation to ensure the absence of supported phylogenetic conflicts. *Elongisporangium anandrum* (Drechsler) Uzuhashi, Tojo and Kakish. was selected as the outgroup taxon for all analyses.

Phylogenetic relationships were inferred using complementary analytical frameworks. Bayesian Inference (BI) was conducted on both single-locus and concatenated datasets of *βtub*, *cox1*, and *cox2* in MrBayes 3.1 via the TrEase web platform [34]. Markov Chain Monte Carlo (MCMC) simulations were run for 10 million generations under a GTR + I + G substitution model, with the initial 25% of trees discarded as burn-in [35]. Maximum Likelihood (ML) analyses were performed in RAxML on TrEase using default parameters, and node support was assessed through 1000 bootstrap replicates. Additionally, Minimum Evolution (ME) analyses were executed on the TrEase server under the Tamura-Nei (TN93) substitution model [36], with clade support assessed using 1000 bootstrap replicates to provide an independent distance-based validation of tree topology and nodal stability. All resulting phylogenetic trees were visualized, annotated, and formatted for publication in MEGA 12 [37]. The sequence alignments and corresponding phylogenetic trees have been deposited in the Zenodo repository (www.zenodo.org; https://www.doi.org/10.5281/zenodo.19588427, accessed on 29 September 2026) to facilitate transparency, accessibility, and reproducibility.

### 2.4. Pathogenicity

Inoculum for fungal isolates was prepared according to the protocol of Banihashemi [38]. For each isolate, 200 g of vermiculite was placed in heat-resistant polypropylene bags, and 120 mL of hempseed extract (extract of 60 g ground hempseed in 1 L of distilled water) was added. The mixture was sterilized three times at 121 °C for 20 min at 24 h intervals. Mycelial inoculum was produced by adding twenty 6 mm mycelial plugs taken from the actively growing edge of three-day-old cultures on CMA to each bag of sterilized vermiculite. Bags were incubated at 25 °C for four weeks, shaken every 4–5 days to ensure uniform colonization. Control inoculum consisted of sterilized vermiculite with hempseed extract plus non-colonized CMA plugs.

To quantify and standardize inoculum levels for pathogenicity tests, a 1 g sample of the substrate was suspended in 10 mL of sterile distilled water and vortexed for one minute. The suspension was filtered through a 50 µm mesh and centrifuged at 3000× *g* for 5 min. The resulting pellet was resuspended in 1 mL of water, and oospore counts were performed with a hemocytometer under a light microscope at 400× magnification. Based on triplicate measurements, the inoculum density was estimated at 50–100 oospores per gram of fresh substrate [39].

Pathogenicity assays were conducted on seedlings of several cucurbit hosts, including cantaloupe, cucumber, muskmelon, squash, and watermelon. For pre-emergence damping-off evaluation, seeds were surface-disinfected with sterile distilled water or 0.5% sodium hypochlorite and dried on sterile paper towels. Infested soil was prepared by mixing inoculum with sterile soil and sand at a 1:10 ratio (soil:sand = 1:1) [40]. Plastic cups (200 mL) were used as pots, with two-thirds of each cup filled with sterile vermiculite. In the remaining upper third, a layer of inoculum was added, followed by the seeds, and then covered with a second layer of inoculum. A total of 5 mL inoculum was applied per pot. Control pots received vermiculite containing hempseed extract but without microbial colonization. For post-emergence damping-off, seedlings were grown for 10 days before inoculation. Soil surrounding the crown was carefully displaced, and 5 mL of the prepared inoculum was applied directly around the crown and root zone before the soil was replaced. Control plants were treated with non-colonized substrate. All pots were maintained under water-logged conditions for 24 h, after which drainage holes were opened to allow excess water to escape. Pots were then transferred to a growth chamber, kept at 25 °C, and irrigated daily with 5 mL of sterile water. Plants were monitored for two weeks following inoculation. The number of healthy and dead seedlings was recorded, and both aerial and root tissues were examined for characteristic symptoms, including wilting, general stunting, and the presence of rot on primary and secondary roots.

Eight plant growth parameters, including root wet weight, root dry weight, foliage wet weight, foliage dry weight, total wet biomass, total dry biomass, root length, and plant height, were meticulously measured after two weeks of inoculation for each plant. Prior to statistical analysis, the distribution of these eight plant growth parameters was evaluated for normality using the Shapiro–Wilk test in IBM SPSS Statistics (version 25; IBM Corp., Armonk, NY, USA). A square root transformation was applied where necessary to correct for skewness [30]. A factorial analysis of variance (ANOVA) within the general linear model (GLM) framework in SAS (version 9.4) (SAS Institute, Cary, NC, USA) was employed to analyze the effects of *Pythium* species, host species, and their interaction on the measured traits. Mean separations were conducted using Tukey’s Honestly Significant Difference (HSD) test at a significance level of *p* ≤ 0.05. Additionally, proportional incidence data (e.g., damping-off percentage and colonization frequency) were validated using Generalized Linear Models (GLMs) with a binomial distribution and logit link function.

Colonization frequency (Cf%) was determined for the roots and crown of each host separately. Inoculated tissues were washed, cut into 0.5 mm segments, surface-dried, and plated (30 segments per 80 mm Petri dish) on CMA-PARP medium. The colonization frequency was calculated using the formula: Cf% = (N_col_/N_t_) × 100, where N_col_ is the number of segments colonized by the target species and N_t_ is the total number of segments cultured [41].

### 2.5. Multivariate and Graphical Analyses of Pathogenicity Traits

Multivariate analyses were conducted to evaluate overall patterns of pathogenicity trait variation among *Pythium* species and cucurbit hosts. Quantitative pathogenicity variables included pre-emergence damping-off incidence (%), crown colonization (%), and root colonization (%). Isolates exhibiting zero values across all evaluated pathogenicity traits were excluded prior to dissimilarity matrix construction to prevent zero-sum mathematical artifacts. Trait values were square-root transformed to reduce the influence of dominant variables while preserving relative differences among isolates.

A non-metric multidimensional scaling (NMDS) ordination was performed using Bray–Curtis dissimilarity matrices implemented in the vegan package (version 2.7.1) [42] in R. NMDS was conducted using the metaMDS function with two dimensions (k = 2), random starting configurations, and a maximum of 999 iterations to ensure convergence on a stable solution. Ordination quality was assessed using the stress value, with stress values < 0.2 considered to provide an acceptable representation of multivariate relationships.

To identify pathogenicity traits most strongly associated with the ordination structure, environmental fitting was performed using the envfit function in vegan with 999 permutations. Pathogenicity traits with significant correlations to the NMDS axes (*p* ≤ 0.05) were visualized as vectors, with arrow length proportional to the strength of the correlation and direction indicating increasing trait values across the ordination space.

Differences in pathogenicity trait composition among *Pythium* species, host species, and their interaction were statistically tested using permutational multivariate analysis of variance (PERMANOVA) based on Bray–Curtis dissimilarities, implemented with the adonis2 function in vegan. To account for the unbalanced design and eliminate order-dependence typical of sequential sums of squares (Type I SS), marginal sums of squares (Type III SS) were evaluated using by = “margin”. Models included species, host, and species × host interaction terms, and significance was assessed using 999 permutations. The proportion of variance explained (R^2^) was calculated for each factor.

The assumption of homogeneity of multivariate dispersion was evaluated prior to the interpretation of PERMANOVA results using the betadisper function, followed by permutation testing. Non-significant dispersion tests (*p* > 0.05) were interpreted as indicating comparable within-group variability and supporting the validity of PERMANOVA inferences. All multivariate analyses and graphical visualizations were performed in R using the vegan, ggplot2 [28], and ggrepel (version 0.9.6) [43] packages.

In addition to multivariate ordination, boxplots were used to visualize trait-specific differences in pathogenicity among species. Pre-emergence damping-off was expressed as incidence (0–1), whereas colonization frequency of roots and crowns was expressed as a percentage. Boxplots display medians and interquartile ranges, with individual isolate values shown to illustrate within-species variability. All NMDS analyses and boxplot visualizations were conducted in R using the packages vegan, ggplot2 [28], dplyr, and tidyr in tidyverse collection [24].

### 2.6. Analysis of Alpha Diversity

Species abundance, defined as the number of isolates per *Pythium* species, was calculated both across the entire dataset and within ecological groupings, including sampling matrix, host, and location. Alpha diversity indices were employed to characterize within-community diversity, comprising species richness (S), the Shannon–Wiener diversity index (H′), the Simpson diversity index (1 − D), the inverse Simpson index (1/D), and Pielou’s evenness index (J′) [44,45]. All analyses were conducted in R environment. The Shannon, Simpson, and inverse Simpson indices were calculated using the diversity function, while species richness was estimated using the specnumber function. Pielou’s evenness index was derived by dividing the Shannon diversity value by the natural logarithm of species richness. The Shannon and Simpson indices were visualized in a single composite figure using grouped bar charts, with ecological matrices displayed on the horizontal axis and alpha diversity values on the vertical axis. Bars representing different diversity indices were placed adjacent to each other within each matrix to facilitate direct comparison. All plots were generated using the ggplot2 package [28], and numerical values of the diversity indices were annotated above the bars to enhance interpretability.

### 2.7. Visualization of Ecological Associations (Sankey Diagram)

To visualize the distribution and ecological associations of *Pythium* species across sampling locations, hosts, and isolation matrices, a Sankey (alluvial) diagram was constructed. The Sankey diagram comprised three categorical axes representing sampling location, host, and isolation matrix, while *Pythium* species were encoded as flow colors. Relative abundances were calculated within each sampling location by normalizing isolate counts to proportions, allowing comparison of species distributions among locations independent of sampling intensity. All visualizations were generated in the R environment using the ggplot2 package [28] in combination with the ggalluvial package (version 0.12.5) [46] for alluvial plotting.

## 3. Results

**Figure 1:**
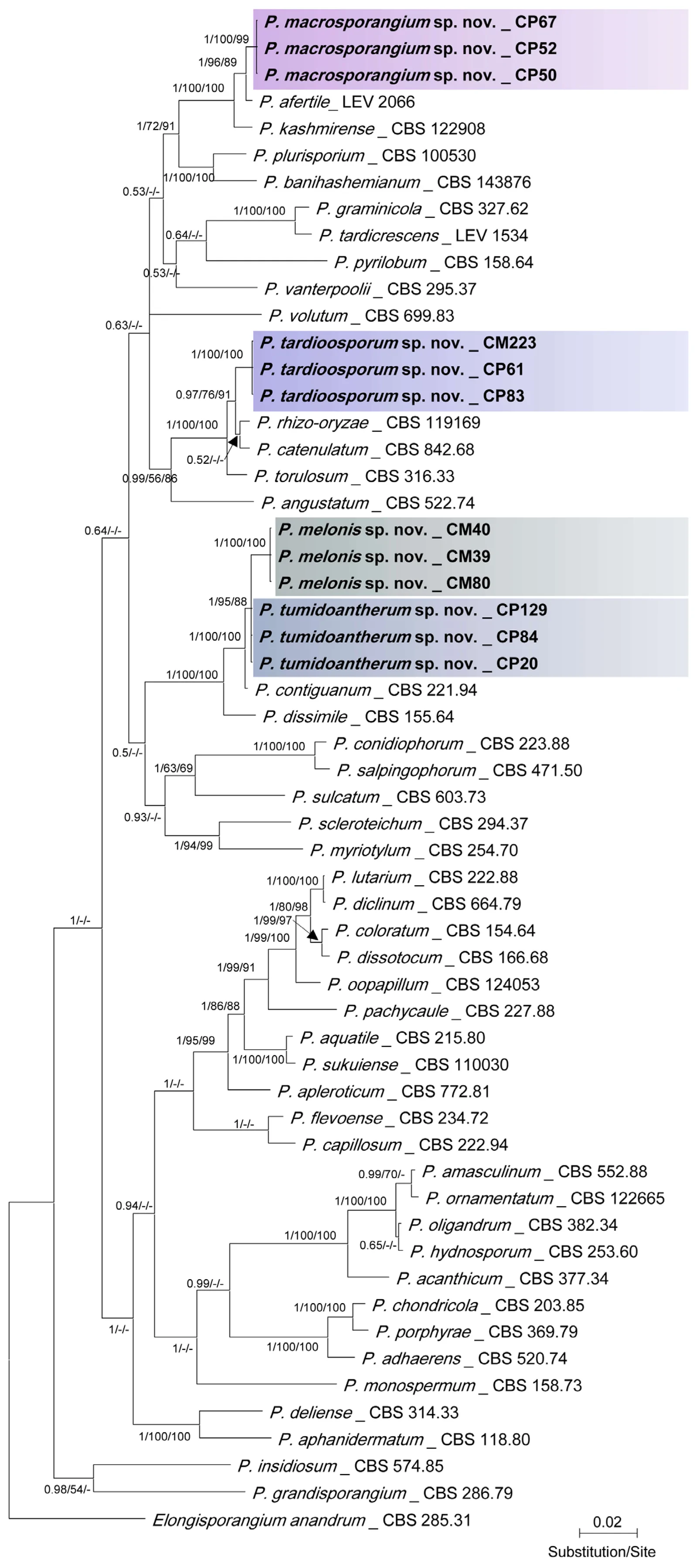
Phylogenetic relationships of novel *Pythium* species from cucurbits and 52 previously described *Pythium* spp. based on Bayesian analyses of a concatenated alignment of nuclear (*βtub*) and mitochondrial (*cox1* and *cox2*) loci. Numbers above or near branches represent Bayesian posterior probabilities, Maximum Likelihood bootstrap support, and Minimum Evolution bootstrap support, respectively, separated by slashes. Values below 50% for bootstrap supports are indicated by hyphens (-).

**Figure 2:**
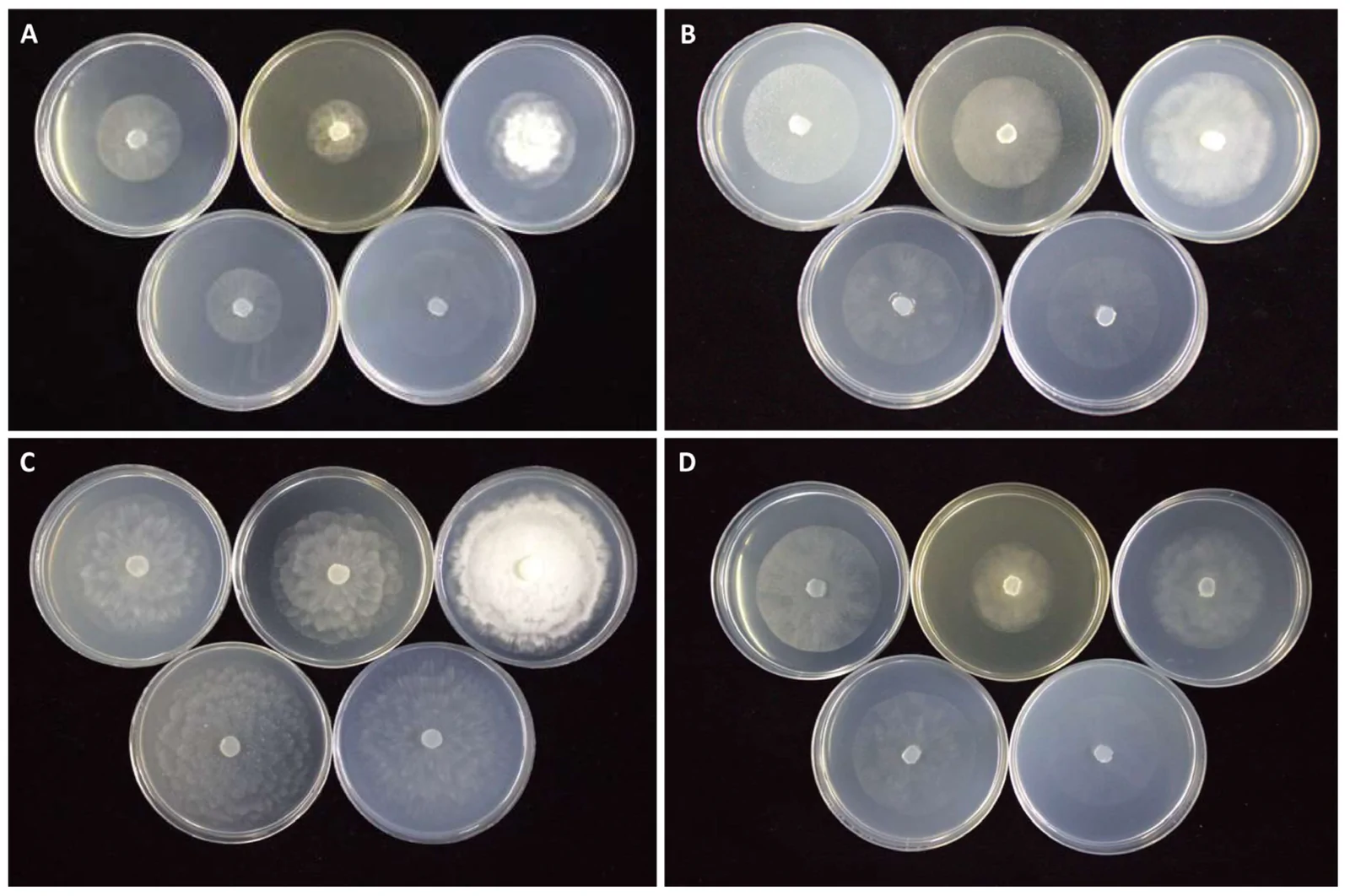
Colony morphology of *Pythium* species from cucurbits on various media at 25 °C. (**A**): *P. macrosporangium* (isolate CP50) after 40 h; (**B**): *P. melonis* (isolate CM39) after 36 h; (**C**): *P. tardioosporum* (isolate CP61) after 36 h; and (**D**): *P. tumidoantherum* (isolate CP20) after 40 h. Top (from left to right): carrot agar, malt extract agar and potato-dextrose agar. Bottom (from left to right): hempseed agar and cornmeal agar.

**Figure 3:**
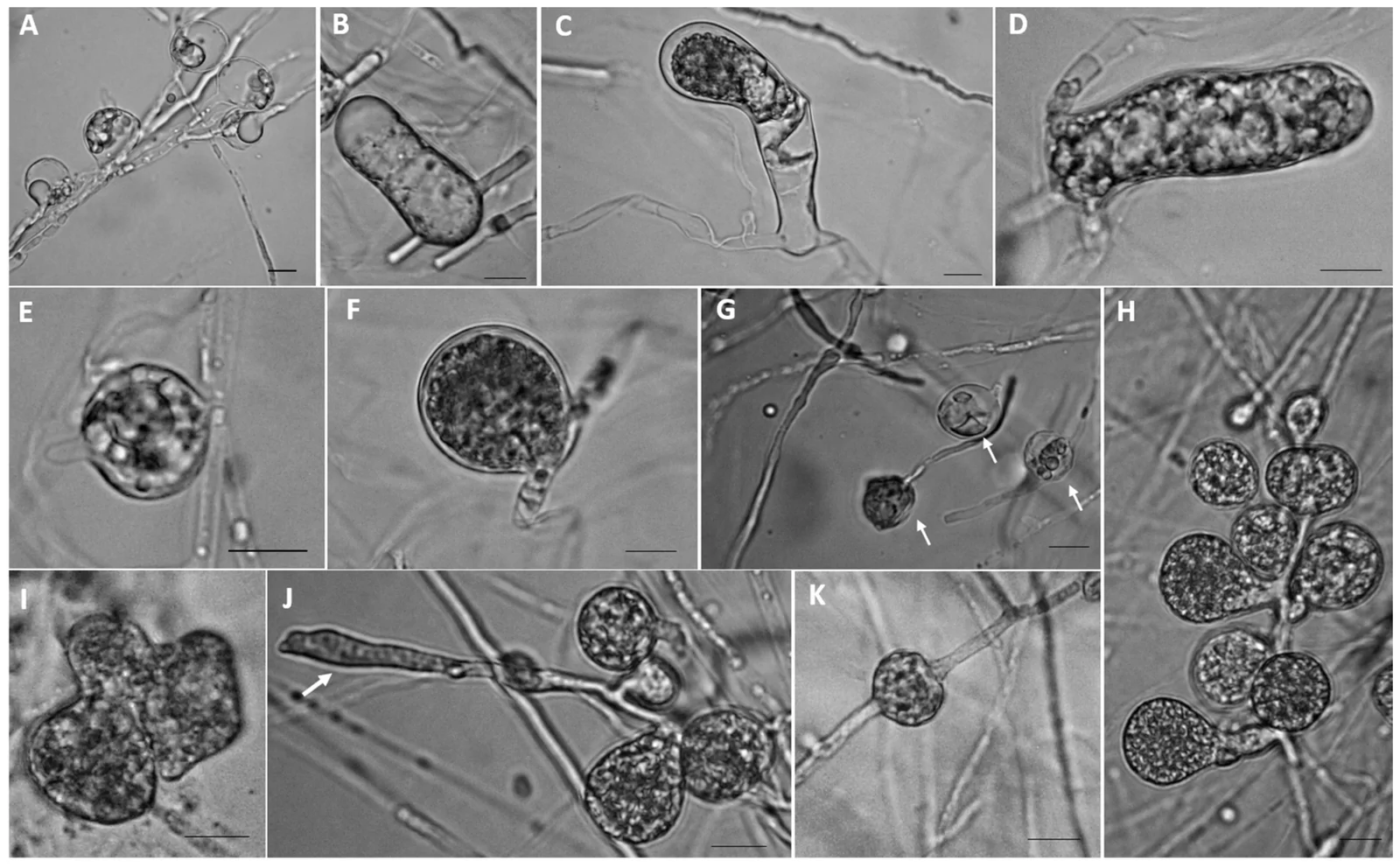
Morphological characteristics of the asexual structures of *Pythium* obtained from cucurbits in this study. (**A**–**F**): *P. macrosporangium*; (**A**): sympodial formation of sporangia; (**B**): irregular appressorium (arrow); (**C**,**D**): irregular intercalary and elongated sporangium; (**E**): globose sporangium with papilla (arrow); (**F**): intercalary hyphal swelling; (**G**): intercalary hyphal swellings (arrows) of *P. melonis*; (**H**–**J**): *P. tardioosporum*; (**H**): hyphal swellings botryoides clustering; (**I**): contiguous sporangia; (**J**): formation of achlyoid structures from hyphal swelling clusters (arrow); (**K**): intercalary hyphal swelling of *P. tumidoantherum* (Bar = 20 µm).

**Figure 4:**
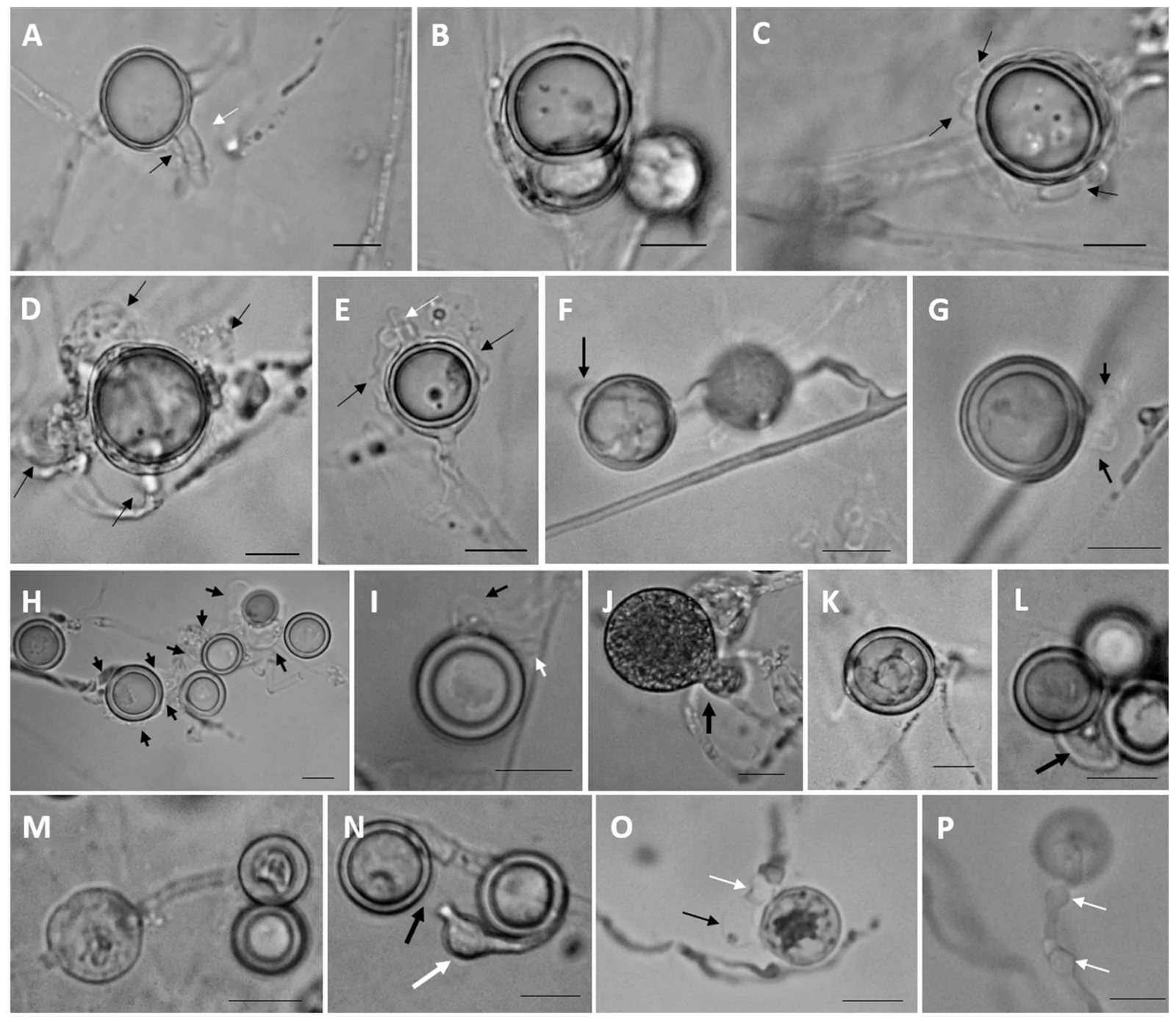
Morphological features of the sexual structures of *Pythium* obtained from cucurbits in this study. (**A**–**E**): *P. macrosporangium* isolate CP50; (**A**): oospore with long papilla (white arrow) and paragynous antheridium (black arrow); (**B**): oogonium with two oospores; (**C**): oospore surrounded by antheridia (arrows); (**D**): aplerotic oospore with up to four giant paragynous antheridia (arrows); (**E**): oospore with large antheridia (black arrows) and long papilla (white arrow); (**F**–**I**): *P. melonis* isolate CM39; (**F**): oospore with sack-shaped antheridium (arrow); (**G**): plerotic oospore with two paragynous antheridia (arrows); (**H**): oospores with up to 4 antheridia per oogonium (arrows); (**I**): oogonium with single crook-necked antheridium (black arrow) and papilla (white arrow); (**J**,**K**): *P. tardioosporum* isolate CP61; (**J**): formation of oospore with paragynous monoclinous antheridium (arrow); (**K**): plerotic mature oospore; (**L**–**P**): *P. tumidoantherum* isolate CP20; (**L**): clustering formation of oospores with large antheridium (arrow); (**M**): catenulated oospores; (**N**): oospore with antheridium (black arrow) and antheridial swellings (white arrow); (**O**): formation of oospore with antheridium (black arrow) and antheridial swelling (white arrow); (**P**): antheridial swellings (white arrows); (Bar = 20 µm).

**Figure 5:**
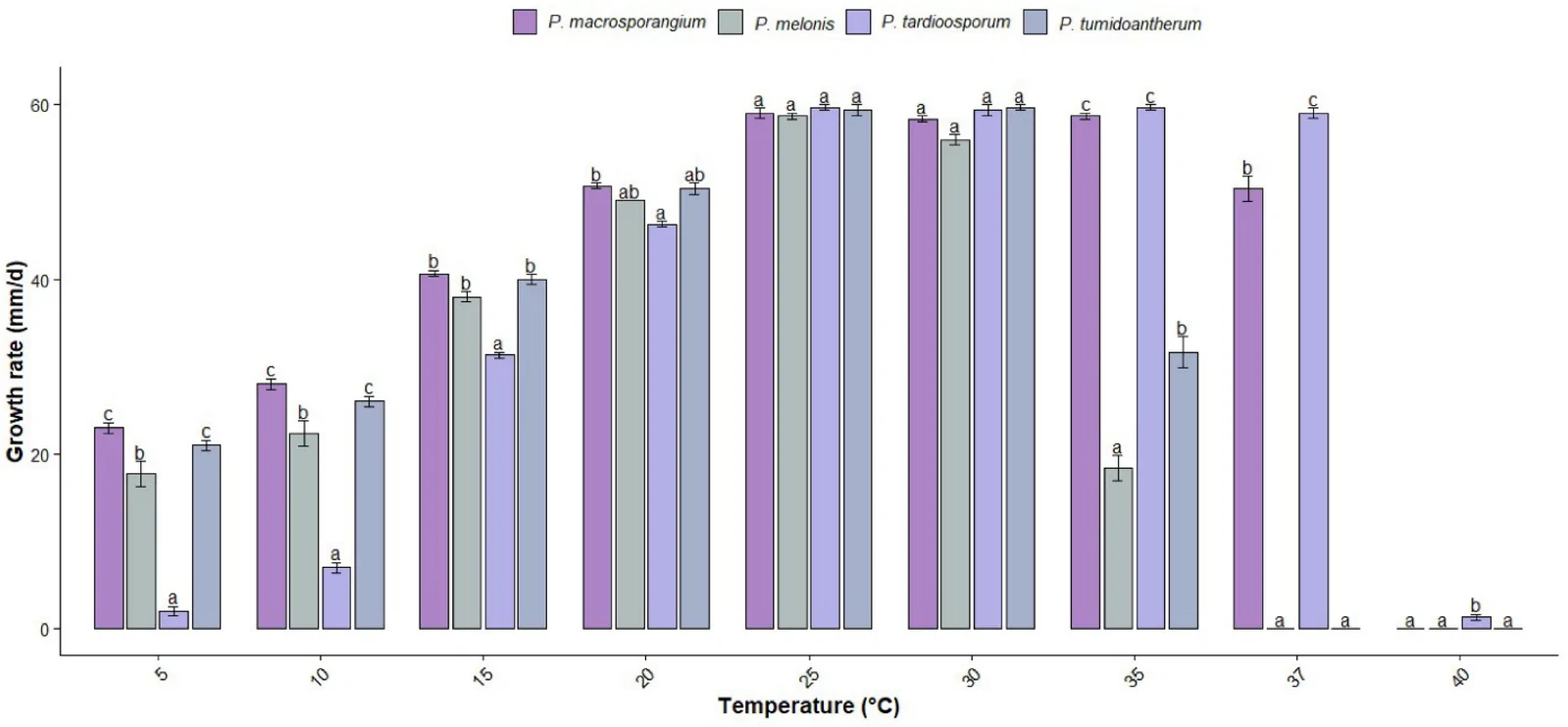
Average radial growth rate of *Pythium macrosporangium* (19 isolates), *P. melonis* (four isolates), *P. tardioosporum* (three isolates), and *P. tumidoantherum* (four isolates) on PCA at different temperatures. Different letters above the bars indicate statistically significant differences among treatments within each temperature, based on Tukey’s honestly significant difference (HSD) test (*p* ≤ 0.05) following two-way ANOVA. Error bars represent ± standard error of the mean (SE).

**Figure 6:**
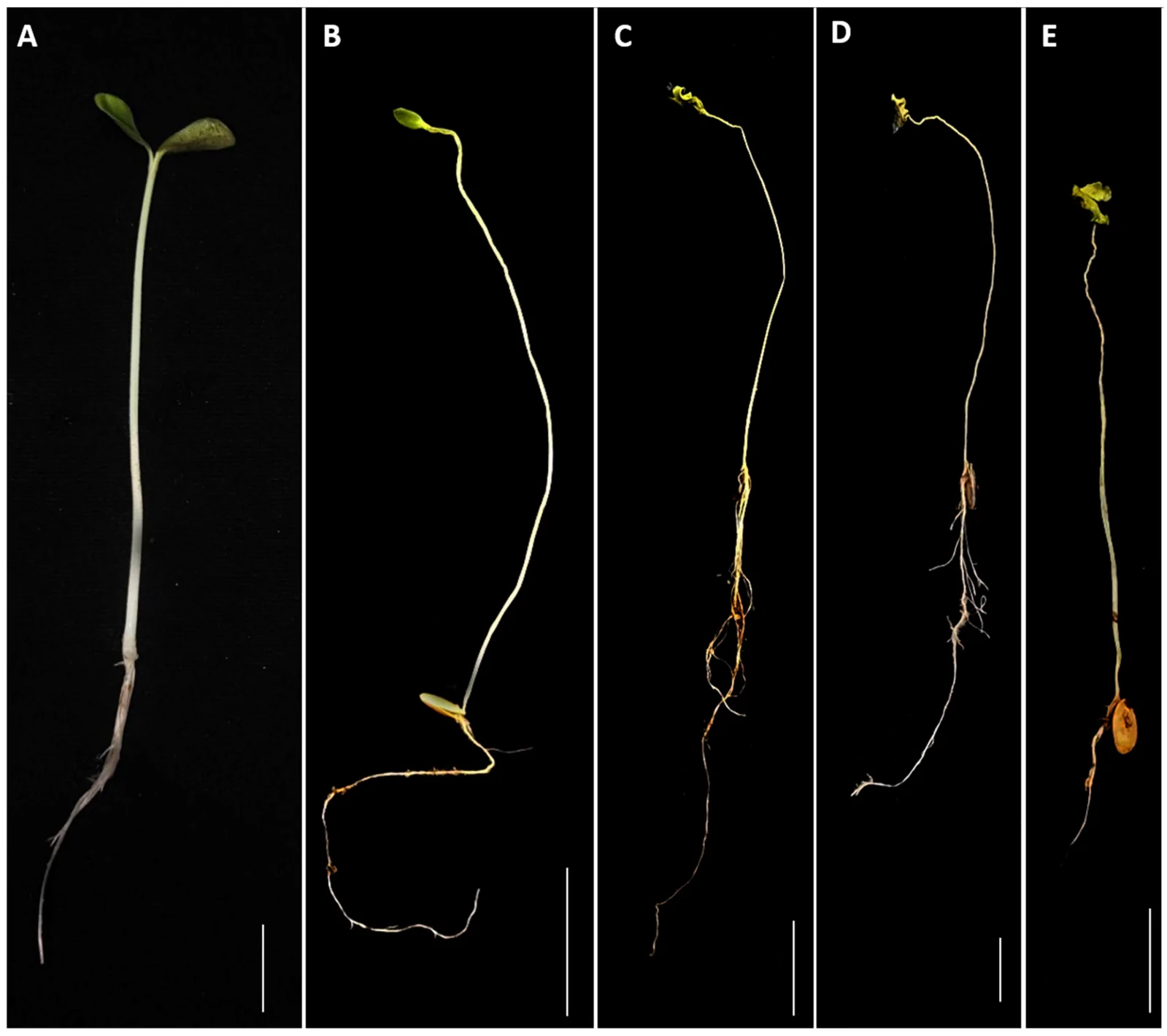
Results of the pathogenicity test of novel *Pythium* species from Clade B on seven-day-old *Cumumis melo* var. *reticulatus* seedlings two weeks after inoculation. (**A**): Control plant; (**B**): severe dwarfing, root rot, and marked reduction in root volume caused by inoculation with *P. macrosporangium*, isolate CP50; (**C**): symptoms of reduced plant growth, root rot, dwarfing, and wilting of aerial parts caused by inoculation with *P. melonis*, isolate CM39; (**D**): severe wilting of aerial parts caused by inoculation with *P. tardioosporum*, isolate CP61; (**E**): symptoms of wilting of aerial parts, root rot, and plant yellowing caused by inoculation with *P. tumidoantherum*, isolate CP20; Bar = 20 mm.

The sampled plants showed severe root and crown rot compared to the healthy ones. A total of 351 oomycete isolates were recovered from root tissue, irrigation water, and rhizosphere soil associated with various cucurbit plants in greenhouses. Of these, 196 were morphologically identified as *Pythium sensu stricto*. Among them, 29 representatives belonging to four morpho-groups within Clade B *sensu* Lévesque and de Cock [5] were selected for further study and are described in this paper (Figure 1; Table 1). These taxa exhibited distinctive morphological characteristics (Figure 2, Figure 3, Figure 4, Figure 5 and Figure 6) and growth rates, as detailed in the Taxonomy section.

Analyses of nuclear (*βtub*) and mitochondrial (*cox1* and *cox2*) loci revealed that the 29 examined isolates from the selected *Pythium sensu stricto* morpho-groups clustered into four distinct phylogenetic lineages (Figure 1 and Figures S1–S3). The final alignment length for *Pythium* spp. isolates was 600 bp for *βtub*, 660 bp for *cox1*, 481 bp for *cox2*, and 1761 bp for the concatenated alignment (Table S4). The sequences of the nuclear and mitochondrial genes were identical among the isolates of each lineage and formed a monophyletic group with maximum support. No significant incongruence was detected among the three loci (*βtub*, *cox1*, and *cox2*), and the datasets were therefore concatenated for subsequent phylogenetic analyses.

All alignments were carefully inspected for anomalies. Protein-coding genes contained no premature stop codons. Phylogenetic analyses of individual and concatenated datasets produced congruent topologies, with all novel species forming well-supported clades (Bayesian posterior probability = 0.95–1.0, Maximum Likelihood and Minimum Evolution bootstrap >80). Multigene genealogical analyses of nuclear and mitochondrial genes showed that the *Pythium* isolates from cucurbits also formed four distinct lineages within Clade B *sensu* Lévesque and de Cock [5], which we designated as *Pythium macrosporangium* sp. nov., *P. melonis* sp. nov., *P. tardioosporum* sp. nov., and *P. tumidoantherum* sp. nov. (Figure 1 and Figures S1–S3).

**Figure 7:**
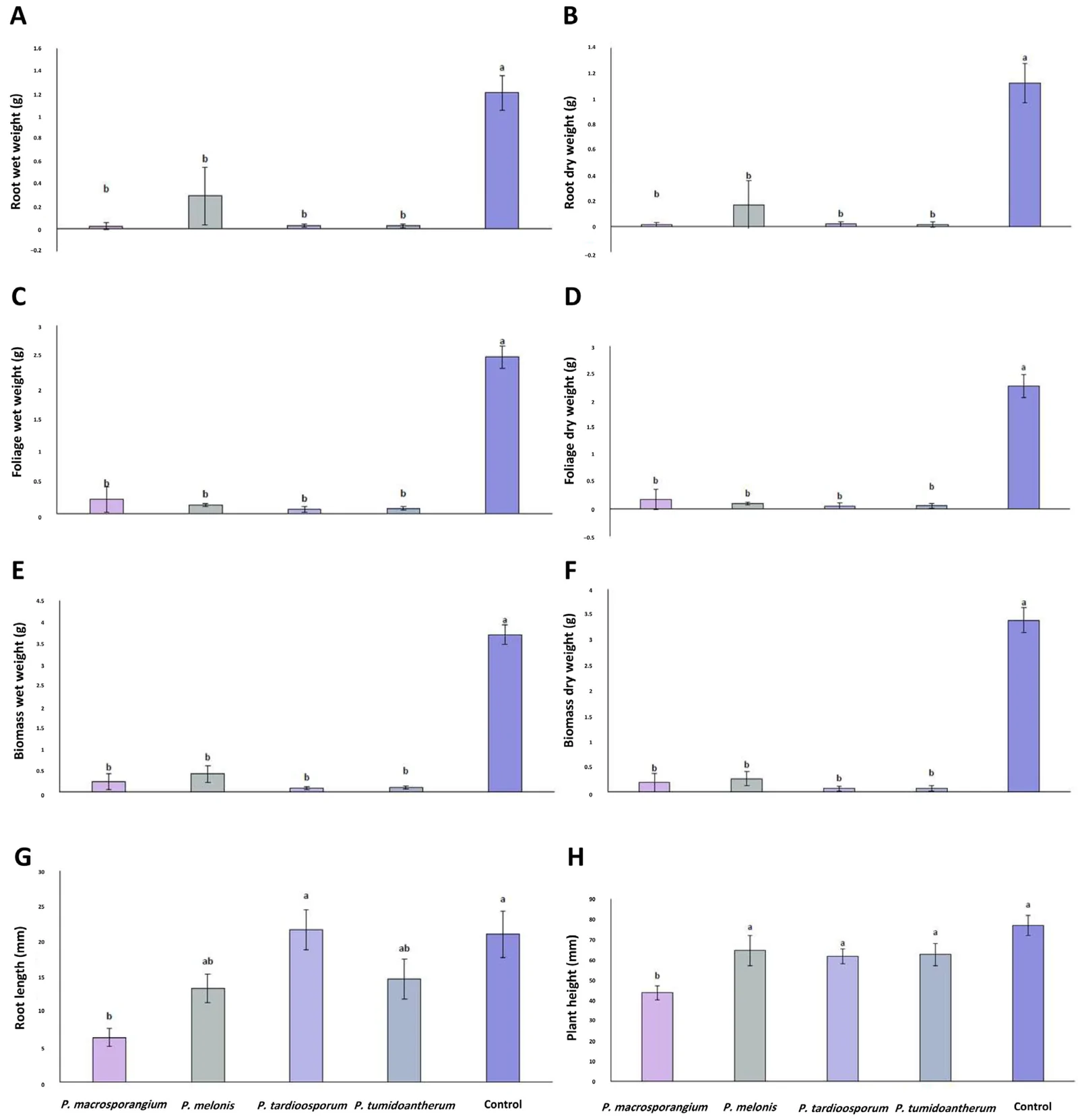
Pathogenic effects of novel *Pythium* species isolated from greenhouse cucurbits in Fars Province on *Cucumis melo* var. *reticulatus*, evaluated based on eight pathogenicity traits and plant growth indices. (**A**): root wet weight; (**B**): root dry weight; (**C**): foliage wet weight; (**D**): foliage dry weight; (**E**): biomass wet weight; (**F**): biomass dry weight; (**G**): root length; (**H**): plant height. Data are presented as mean ± standard deviation (SD). Error bars represent standard deviation of the replicates, and different letters indicate statistically significant differences among treatments at *p* < 0.05.

Temperature, species, and their interaction had highly significant effects on the mycelial growth of *Pythium* Clade B isolates from four morpho-groups. The two-way ANOVA conducted on square-root-transformed data revealed a strong main effect of species and temperature, as well as a highly significant species × temperature interaction (Figure 7; Table S5). Across all isolates, mycelial growth increased with temperature from 5 to 25 °C, reaching maximum values between 25 and 30 °C, followed by a decline at higher temperatures. Growth was markedly reduced at 37 °C and nearly absent at 40 °C for most isolates, although notable differences among treatments were observed under high-temperature conditions. At lower temperatures (5–10 °C), isolates differed significantly in growth, with some Clade B isolates (i.e., *P. tardioosporum*) exhibiting minimal growth while others maintained moderate mycelial expansion (Figure 7). Post hoc Tukey HSD comparisons within each temperature level confirmed that species effects were temperature dependent. At intermediate temperatures (15–30 °C), several isolates did not differ significantly from one another. In particular, one group (i.e., *P. melonis*) maintained comparatively high growth at 35–37 °C, while others showed significant growth suppression.

### 3.1. Taxonomy

***Pythium macrosporangium*** Salmanin., Sekandarpour, and Mostowf. **sp. nov.** (Figure 1, Figures S1–S3, Figure 2A, Figure 3A–F, Figure 4A–E and Figure 5)

Mycobank: 863434

*Type*: IRAN, Fars Province: Arsanjan, from the rhizosphere soil of *Cucurbita pepo*, October 2025, F. Salmaninezhad, CP50, GenBank: *cox1* = PX985171, *cox2* = PZ277340, *βtub* = PZ305329, (holotype IRAN 5682C, preserved in a metabolically inactive state, Iranian National Fungi Collection, Tehran, Iran).

*Etymology*: The name refers to the organism’s production of large sporangia.

*Description*: *Main hyphae*: up to 4.8 µm wide. *Colonies*: on PDA with thick cottony mycelia forming a median pattern, on CMA with radial pattern, on CA with stellate pattern, on MEA and HSA with rosette pattern (Figure 2A). *Sporangia*: irregular, sack-shaped, obovoid, obpyriform, elongated, occasionally catenulated, 120 (71–153.1) × 32 (17.5–41.3) µm diam., may bear a papilla up to 4 µm long (Figure 3E). *Hyphal swellings*: irregular, sack-shaped, obovoid, subglobose, obpyriform, elongated, 70 (40.5–91.5) × 27 (12.3–32.1) µm diam. *Zoospores and Vesicles*: rarely formed after 96 h at dark at 22 °C. Chlamydospores: rarely formed, terminal, globose, 21 (9.9–32) µm diam. *Appressoria*: irregular to sack-shaped, only formed on solid media. *Homothallic*. *Oogonia*: globose, 25.6 (22–47) µm diam., subglobose, smooth, terminal, occasionally intercalary (Figure 4A–E), with a papilla up to 19 µm long (Figure 4A,E). *Antheridia*: sack-shaped to club-shaped, 23 (17–32) × 18 (11–21) µm, 1–4 per oogonium, paragynous, rarely amphigynous (<2%), monoclinous to diclinous, mostly surrounding the oogonium (Figure 4A–E). *Oospores*: globose, subglobose, 22.6 (19–43) µm diam., 1–2 per oogonium, aplerotic, with a wall up to 2.7 µm width. *Cardinal temperatures*: minimum 5 °C, optimum 35 °C, and maximum 37 °C. Daily growth rate at 25 °C on PCA was 25.8 mm/d. Morphological features of this species and its comparison with other known species, as well as other Clade B members in this study is shown in Table 2 and Table S3.

*Notes*: This species is located at Clade B of *Pythium sensu stricto sensu* Lévesque and de Cock [5] in the vicinity of *P. afertile* Kanouse and Humphrey and *P. kashmirense* Paul (Figure 1 and Figures S1–S3; Table S4).

*Other specimens examined*: IRAN, Fars Province, Beyza, from diseased roots of *Cu. pepo*, October 2025, F. Salmaninezhad, CP52, GenBank: *cox1* = PZ277324, *cox2* = PZ277341, *βtub* = PZ305330. IRAN, Fars Province, Shiraz, from irrigation water of *Cu. pepo* greenhouse, December 2025, F. Salmaninezhad, CP67, GenBank: *cox1* = PZ277325, *cox2* = PZ277342, *βtub* = PZ305331. IRAN, Fars Province, Shiraz, from irrigation water of *Cu. pepo* greenhouse, December 2025, F. Salmaninezhad, CP102. IRAN, Fars Province, Marvdasht, from irrigation water of *Cu. pepo* greenhouse, December 2025, F. Salmaninezhad, CP104. IRAN, Fars Province, Dasht-e-Arjan, from the rhizosphere soil of *Cu. pepo,* December 2025, F. Salmaninezhad, CP117. IRAN, Fars Province, Kazerun, from the rhizosphere soil of *Cu. pepo,* December 2025, F. Salmaninezhad, CP202. IRAN, Fars Province, Dasht-e-Arjan, from the rhizosphere soil of *Cu. pepo,* December 2025, F. Salmaninezhad, CP107. IRAN, Fars Province, Neyriz, from the rhizosphere soil of *Cu. pepo,* December 2025, F. Salmaninezhad, CP80. IRAN, Fars Province, Fasa, from the rhizosphere soil of *C. sativus*, December 2025, F. Salmaninezhad, CS112. IRAN, Fars Province, Kazerun, from irrigation water of *Cu. pepo* greenhouse, December 2025, F. Salmaninezhad. IRAN, Fars Province, Neyriz, from irrigation water of *Cu. pepo* greenhouse, December 2025, F. Salmaninezhad, CP476. IRAN, Fars Province, Arsanjan, from irrigation water of *Cu. pepo* greenhouse, December 2025, F. Salmaninezhad, CP477. IRAN, Fars Province, Beyza, from irrigation water of *Cu. pepo* greenhouse, December 2025, F. Salmaninezhad, CP479. IRAN, Fars Province, Arsanjan, from irrigation water of *Cu. pepo* greenhouse, December 2025, F. Salmaninezhad, CP481. IRAN, Fars Province, Sarvestan, from the rhizosphere soil of *Cu. pepo,* December 2025, F. Salmaninezhad, CP482. IRAN, Fars Province, Beyza, from the rhizosphere soil of *Cu. pepo,* December 2025, F. Salmaninezhad, CP485. IRAN, Fars Province, Shiraz, from the diseased roots of *C. melo* var. reticulatus, December 2025, F. Salmaninezhad, CM333. IRAN, Fars Province, Beyza, from the rhizosphere soil of *Cu. pepo,* December 2025, F. Salmaninezhad, CP349.

***Pythium melonis*** Mostowf., Sekandarpour and Salmanin. **sp. nov.** (Figure 1, Figures S1–S3, Figure 2B, Figure 3F, Figure 4F–I and Figure 5)

Mycobank: 863435

*Type*: IRAN, Fars Province: Arsanjan, on the diseased roots of *Cucumis melo* var. *reticulatus*, October 2025, F. Salmaninezhad, CM39, GenBank: *cox1* = PX985154, *cox2* = PZ277331, *βtub* = PZ305320, (holotype CBS 155836, preserved in a metabolically inactive state, Westerdijk Fungal Biodiversity Institute, Utrecht, The Netherlands).

*Etymology*: Named after *Cucumis melo* var. *reticulatus*, the host plant from which the species was isolated.

*Description*: *Main hyphae*: up to 5 µm wide. *Colonies*: on CMA and CA with a radial pattern, on PDA and HSA with a rosette pattern, and on MEA with a median pattern (Figure 2B). *Sporangia*: strictly filamentous, not differentiated from the main hyphae. *Zoospores and Vesicles*: rarely formed at 20 °C after 72 h. *Hyphal swellings*: globose, subglobose, 20 (15.9–23.1) µm diam., mostly intercalary (Figure 3F). Appressoria: absent. Homothallic. *Oogonia*: globose, 30.5 (26.1–32.6) µm diam., smooth, rarely papillated with a papilla up to 3 µm long (5.7%), rarely formed in chains (2.1%) (Figure 4F–I). *Antheridia*: rarely seen, club-shaped, sack-shaped, 13.2 (9.6–15.4) × 9.8 (7.2–11.9) µm, 1–5 per oogonium, mostly diclinous, occasionally monoclinous, paragynous. (Figure 4F–I). *Oospores*: one per oogonium, perfectly plerotic, 30.5 (26.1–32.6) µm diam., with a wall up to 3.3 µm width (Figure 4F–I). *Cardinal temperatures*: minimum 5 °C, optimum 30 °C, and maximum 35 °C. Daily growth rate at 25 °C on PCA was 27.7 mm/d (Figure 5). Morphological features of this species and its comparison with other known species, as well as other clade B members in this study is shown in Table 2 and Table S3.

*Notes*: This species is located at Clade B of *Pythium sensu stricto sensu* Lévesque and de Cock [5] in the vicinity of *P. contiguanum* Paul (Figure 1 and Figures S1–S3; Table S4).

*Other specimens examined*: IRAN, Fars Province, Arsanjan, from diseased roots of *C. melo* var. *reticulatus*, October 2025, F. Salmaninezhad, CM40, GenBank: *cox1* = PX985155, *cox2* = PZ277332, *βtub* = PZ305321. IRAN, Fars Province, Shiraz, from diseased roots of *C. melo* var. *reticulatus*, December 2025, F. Salmaninezhad, CM442. IRAN, Fars Province, Marvdasht, from diseased roots of *C. melo* var. *reticulatus*, October 2025, F. Salmaninezhad, CM80, GenBank: *cox1* = PX985156, *cox2* = PZ277333, *βtub* = PZ305322.

***Pythium tardioosporum*** Salmanin., Sekandarpour, and Mostowf. **sp. nov.** (Figure 1, Figures S1–S3, Figure 2C, Figure 3H–J, Figure 4J–K and Figure 5)

Mycobank: 863436

*Type*: IRAN, Fars Province: Beyza, from the rhizosphere soil of *Cucurbita pepo*, November 2025, F. Salmaninezhad, CP61, GenBank: *cox1* = PX985160, *cox2* = PZ277337, *βtub* = PZ305326, (holotype IRAN 5681C, preserved in a metabolically inactive state, Iranian National Fungi Collection, Tehran, Iran).

*Etymology*: The name refers to prolonged production of oospores.

*Description*: *Main hyphae*: up to 4.7 µm wide. *Colonies*: on PDA with thick cottony mycelia forming a median pattern, on MEA with petaloid pattern, on CA and HSA with a rosette pattern, and on CMA with a chrysanthemum pattern (Figure 2C). *Sporangia*: filamentous, inflated, contiguous, forming lobate structures (Figure 3I). *Zoospores and Vesicles*: rarely formed at 22 °C after 18 h. *Hyphal swellings*: globose, subglobose, irregular shapes, 35 (19.5–41) µm diam., mostly intercalary, formed in botryoides clustering (Figure 3H), and more than 70% with achloyoid structures at the end of clustering (Figure 3J). *Appressoria*: only formed on solid medium, fusiform. *Homothallic*. Sexual structures rarely formed after 90 d on CA. *Oogonia*: globose, terminal, smooth, 39 (21.5–50.3) µm diam., never formed in chains (Figure 4J–K). *Antheridia*: crook-necked, 11.2 (7.6–14.4) × 7.7 (5.2–10.3) µm, one per oogonium, rarely diclinous, mostly monoclinous, rarely diclinous (3.4%), paragynous (Figure 4J). *Oospores*: one per oogonium, mostly plerotic, 39 (21.5–50.3) µm diam., with a wall up to 2.9 µm width. *Cardinal temperatures*: minimum 5 °C, optimum 25 °C, and maximum 40 °C. Daily growth rate at 25 °C on PCA was 28 mm/d. Morphological features of this species and its comparison with other known species, as well as other clade B members in this study is shown in Table 2 and Table S3.

*Notes*: This species is located at Clade B of *Pythium sensu stricto sensu* Lévesque and de Cock [5] in the vicinity of *P. catenulatum* and *P. rhizo-oryzae* (Figure 1 and Figures S1–S3; Table S4).

*Other specimens examined*: IRAN, Fars Province, Dasht-e-Arjan, from diseased roots of *Cu. pepo*, December 2025, F. Salmaninezhad, CP83, GenBank: *cox1* = PX985161, *cox2* = PZ277339, *βtub* = PZ305328. IRAN, Fars Province, Fasa, from diseased roots of *C. melo* var. *reticulatus*, November 2025, F. Salmaninezhad, CM223, GenBank: *cox1* = PX985162, *cox2* = PZ277338, *βtub* = PZ305327.

***Pythium tumidoantherum*** Mostowf., Sekandarpour, and Salmanin. **sp. nov.** (Figure 1, Figures S1–S3, Figure 2D, Figure 3K, Figure 4L–P and Figure 5)

Mycobank: 863437

*Type*: IRAN, Fars province: Beyza, on the diseased roots of *Cucurbita pepo*, October 2025, F. Salmaninezhad, CP20, GenBank: *cox1* = PX985157, *cox2* = PZ277334, *βtub* = PZ305323, (holotype IRAN 5680C, preserved in a metabolically inactive state, Iranian National Fungi Collection, Tehran, Iran).

*Etymology*: The name refers to the swollen, chain-like antheridial stalk before attaching to the oogonium.

*Description*: *Main hyphae*: up to 3.1 µm wide. *Colonies*: on CMA and CA with a radial pattern, on PDA and MEA with a rosette pattern, and on HSA with a chrysanthemum pattern (Figure 2D). *Sporangia*: absent. *Zoospores and Vesicles*: not formed. *Hyphal swellings*: rarely formed after 10 d on CA, subglobose, intercalary, 9.9 (4.6–15.7) µm diam (Figure 3K). *Appressoria*: absent. *Homothallic*. *Oogonia*: globose, subglobose to amorphous, mostly intercalary, rarely terminal (10.2%), smooth, 17.2 (13.1–22.5) µm diam., produced in continuous form from a single hypha, clustering, catenulated (Figure 4L–N). *Antheridia*: fusiform, 1–3 per oogonium, diclinous, paragynous, rarely hypogynous (11.1%), attached laterally or terminally to the oogonium, antheridial stalk with chain-like swellings before attachment to the oogonium (Figure 4O–P). *Oospores*: mostly aborted, one per oogonium, plerotic, 17.2 (13.1–22.5) µm diam., with a wall up to 1.5 µm width. *Cardinal temperatures*: minimum 5 °C, optimum 30 °C, and maximum 35 °C. Daily growth rate at 25 °C on PCA was 28.1 mm/d. Morphological features of this species and its comparison with other known species, as well as other Clade B members in this study is shown in Table 2 and Table S3.

*Notes*: This species is located at Clade B of *Pythium sensu stricto sensu* Lévesque and de Cock [5] in the vicinity of *P. contiguanum*. This species is also closely to the novel species described in this study, herein designated as *P. melonis* sp. nov. (Figure 1 and Figures S1–S3; Table S4).

*Other specimens examined*: IRAN, Fars Province, Neyriz, from diseased roots of *Cu. pepo*, December 2025, F. Salmaninezhad, CP84, GenBank: *cox1* = PX985158, *cox2* = PZ277336, *βtub* = PZ305325. IRAN, Fars Province, Shiraz, from diseased roots of *Cu. pepo,* December 2025, F. Salmaninezhad, CP129, GenBank: *cox1* = PX985159, *cox2* = PZ277335, *βtub* = PZ305324. IRAN, Fars province, Marvdahst, from the rhizosphere soil of *Cu. pepo,* December 2025, F. Salmaninezhad, CP801.

### 3.2. Pathogenicity Tests

Non-inoculated control plants treated with sterile substrate remained completely symptom-free throughout the trial period, with no evidence of pre-emergence damping-off, root necrosis, or stunting (Figure 6A), and no oomycetes were re-isolated from control root or crown tissues. In contrast, pathogenicity tests showed that all novel *Pythium* species caused disease on *C. melo* var. *reticulatus*. Among the other tested plant species and varieties, pathogenicity varied according to the oomycete species. *Pythium tumidoantherum* colonized the roots of *Cu. pepo* and caused pre-emergence damping-off. In addition, *P. macrosporangium* colonized the root and crown tissues of *Ci. lanatus*, *C. melo* var. *cantalupensis*, *C. melo* var. *reticulatus*, and *Cu. pepo* and caused pre-emergence damping-off. In *C. melo* var. *reticulatus*, all tested isolates caused severe root rot and significantly reduced root fresh and dry weights, shoot fresh and dry weights, and root length (Figure 6 and Figure 7; Table S7).

#### 3.2.1. Multivariate Analysis of Pathogenicity Traits

**Figure 8:**
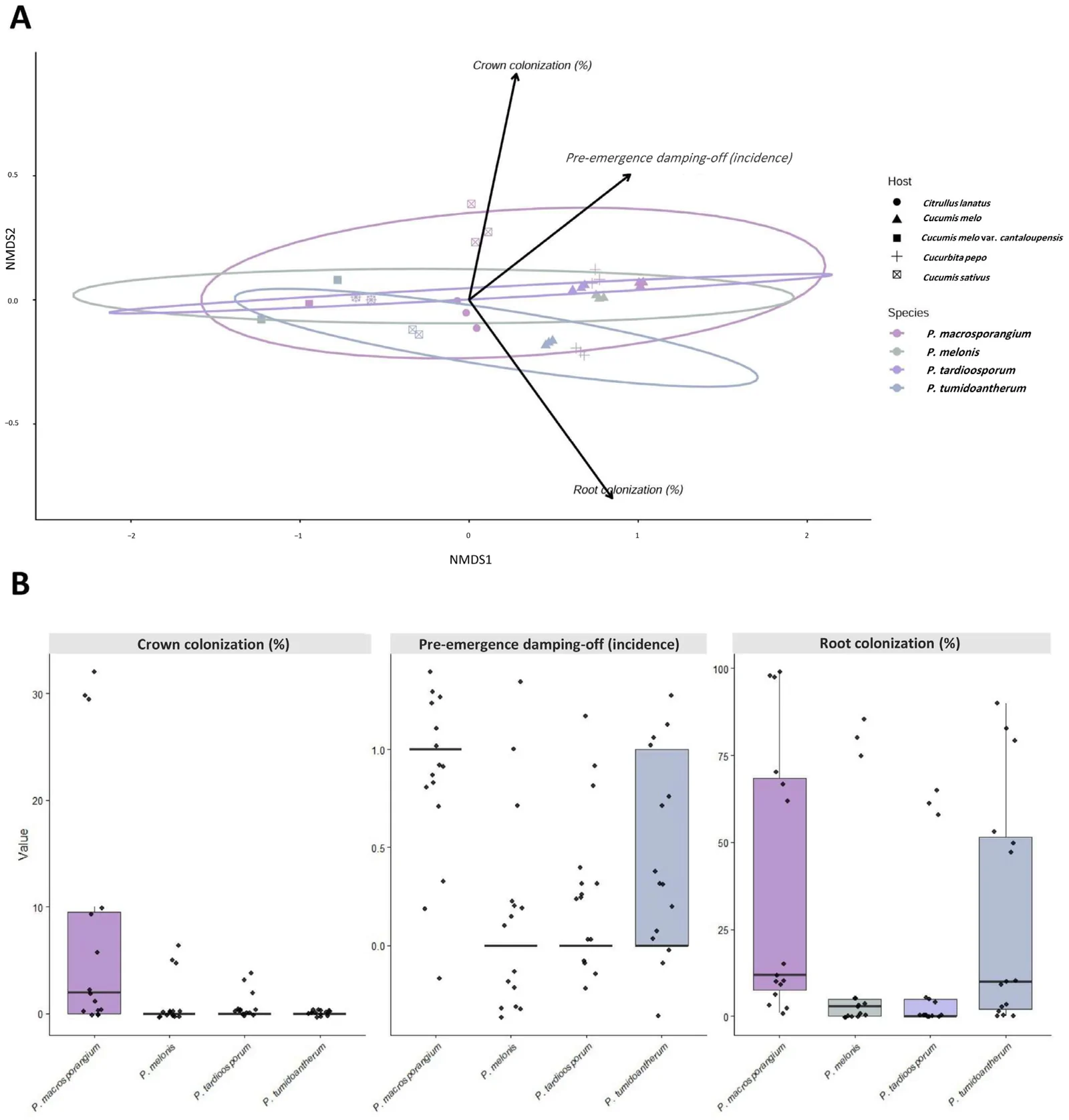
Pathogenicity variation among *Pythium* species in this study. (**A**): Non-metric multidimensional scaling (NMDS) ordination of pathogenicity traits based on Bray–Curtis dissimilarities. Points represent individual isolates and are colored by host and shaped by species. Ellipses indicate 95% confidence intervals for host groups. Arrows represent pathogenicity traits significantly associated with the ordination as determined by envfit (*p* ≤ 0.05), with arrow length proportional to the strength of correlation. The NMDS solution showed a low stress value (stress = 0.11). (**B**): Distribution of pathogenicity traits among species. Boxplots show median and interquartile range, with points representing individual isolates. Pre-emergence damping-off is shown as incidence (0–1), whereas root and crown colonization are expressed as percentages.

Bray–Curtis–based multivariate analyses revealed clear structure in pathogenicity traits among *Pythium* spp. isolates (Figure 8A). The non-metric multidimensional scaling (NMDS) ordination produced a stable two-dimensional solution with a final stress value of approximately 0.11. The ordination showed pronounced separation among host species and more moderate differentiation among *Pythium* species. Permutational multivariate analysis of variance (PERMANOVA) incorporating species, host, and their interaction detected a highly significant species × host interaction effect (Table S6). This interaction explained 16.0% of the total variation in pathogenicity trait composition (F_6_,_28_ = 26.164, *p* = 0.001). When marginal effects were examined, both species and host independently contributed significantly to multivariate variation. Species identity explained 16.2% of the variance (F_3_,_34_ = 9.704, *p* = 0.001), whereas host identity accounted for the largest proportion of variation, explaining 74.9% of the total variance in pathogenicity traits (F_4_,_34_ = 33.687, *p* = 0.001). The order in which species and host were entered into the model did not affect the magnitude or significance of their marginal effects. Tests of homogeneity of multivariate dispersion indicated no significant differences in within-group variability among species or hosts. Dispersion did not differ among Pythium species (F_3_,_38_ = 0.042, *p* = 0.988) or among host species (F_4_,_37_ = 1.993, *p* = 0.116).

#### 3.2.2. Trait-Specific Pathogenicity Patterns

Boxplot analyses revealed marked differences among *Pythium* species in colonization frequency and pre-emergence damping-off incidence (Figure 8B). Crown colonization (%) was highest and most variable in *P. macrosporangium*, whereas *P. melonis*, *P. tardioosporum*, and *P. tumidoantherum* exhibited consistently low crown colonization values. Root colonization (%) also differed among species, with *P. macrosporangium* showing the highest median values and widest dispersion, followed by *P. tumidoantherum*, while *P. melonis* and *P. tardioosporum* generally showed low colonization frequencies. Pre-emergence damping-off incidence displayed a binary response pattern (0–1) across species. *Pythium macrosporangium* and *P. tumidoantherum* exhibited higher and more variable incidence among isolates, whereas damping-off incidence was low or absent for most isolates of *P. melonis* and *P. tardioosporum*. Collapsed interquartile ranges observed for some species reflected the discrete nature of the incidence data.

### 3.3. Species Composition and Alpha Diversity Results

**Figure 9:**
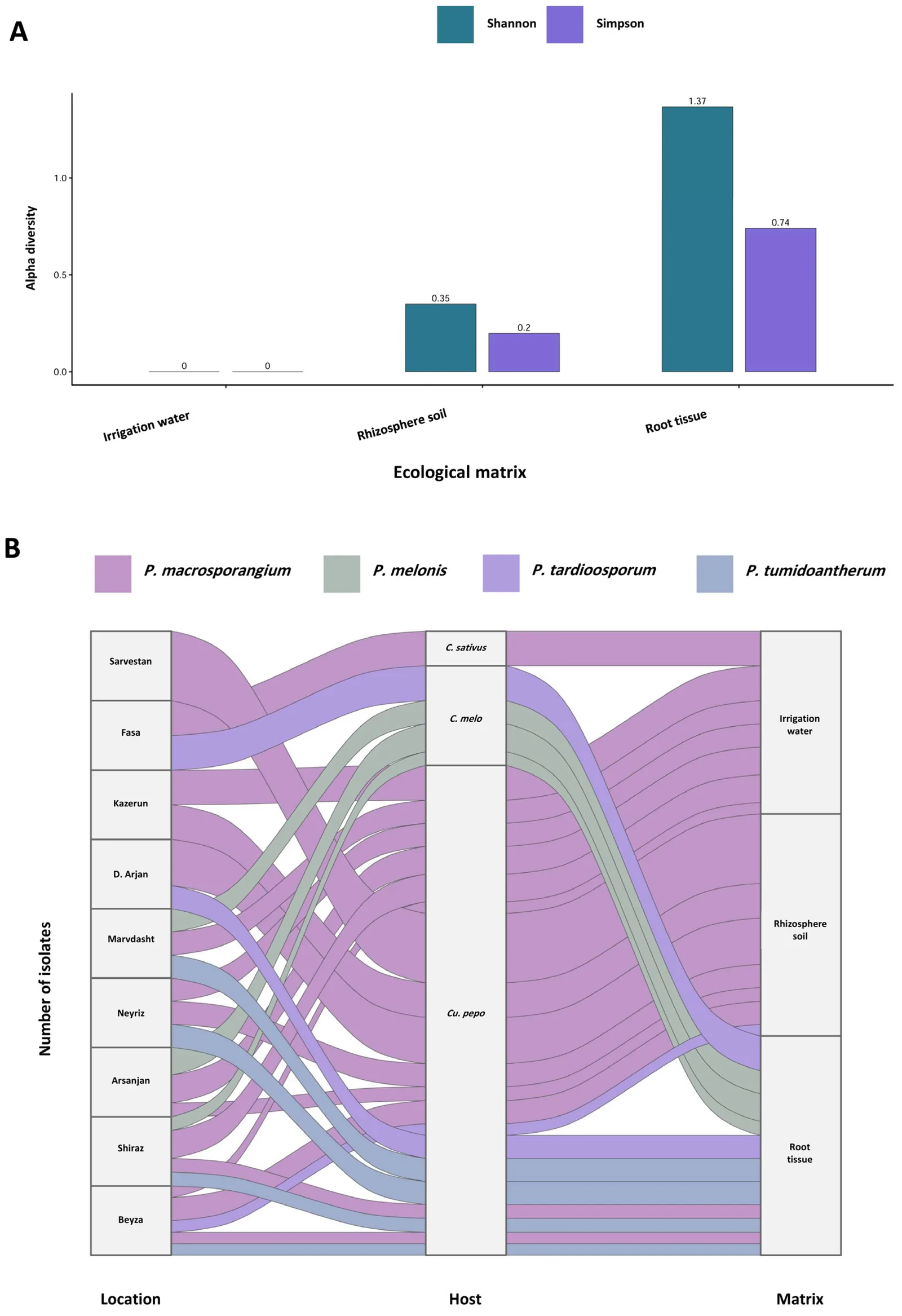
Alpha diversity and ecological distribution of novel *Pythium* species from Clade B across different ecological substrates. (**A**): Shannon diversity (H′) and Simpson diversity (1 − λ) indices are shown for irrigation water, rhizosphere soil, and root tissues. (**B**): Sankey (alluvial) diagram illustrating the distribution of *Pythium* species across sampling locations, host plants (*Cucumis melo* var. *reticulatus*, *C. sativus*, and *Cucurbita pepo*), and ecological matrices. The width of each flow is proportional to the number of isolates, and colors denote individual *Pythium* species.

Among the four taxa recovered in this study, community structure was numerically dominated by *P. macrosporangium*. This taxon was detected across all hosts, locations, and ecological substrates examined. Analysis of alpha diversity revealed pronounced differences among ecological substrates (Figure 9A). A total of N = 9 isolates were recovered from irrigation water (representing 47.4% of the 19 total *P. macrosporangium* isolates obtained in this study). All nine aquatic isolates belonged exclusively to *P. macrosporangium*, resulting in a species richness of one (S = 1) and consequently zero values for both the Shannon (H′ = 0) and Simpson (1 − λ = 0) diversity indices under the culture-dependent framework. Rhizosphere soil displayed slightly higher, yet still limited, diversity, with a species richness of two and low values for the Shannon (H′ = 0.349) and Simpson (1 − λ = 0.198) indices. Although more than one taxon was recovered from this substrate, *P. macrosporangium* remained dominant, while the other taxa occurred only sporadically. In contrast, root tissues exhibited the highest level of alpha diversity among all substrates. All four identified taxa were isolated from this substrate, resulting in a species richness of four, a relatively high Shannon diversity index (H′ = 1.33), and substantial evenness, as reflected by the Simpson index (1 − λ = 0.72). Overall, alpha diversity in irrigation water was zero (Shannon H′ = 0; Simpson 1 − λ = 0). Diversity increased in the rhizosphere soil and reached its maximum in root tissues (Figure 9A).

The Sankey diagram (Figure 9B) illustrates the distribution of *Pythium* species across sampling locations, hosts, and ecological substrates. Consistent with alpha diversity patterns (Figure 9A), *P. macrosporangium* dominated all ecological pathways, forming the largest flows across all hosts and locations. This species was the sole taxon associated with irrigation water and contributed the majority of isolates recovered from rhizosphere soil and root tissues. In contrast, the remaining taxa exhibited narrower and more compartmentalized flows, being largely restricted to root tissues and specific host–location combinations.

## 4. Discussion

This study provides an integrative and detailed characterization of novel oomycete diversity associated with cucurbit greenhouse systems and substantially refines the current understanding of species boundaries, character evolution, and ecological relevance within *Pythium sensu stricto*, particularly Clade B. Through extensive sampling of irrigation water, rhizosphere soil, and root tissues, and the analysis of the novel *Pythium* isolates, we demonstrated that greenhouse environments support a complex and structured *Pythium* community that has been underestimated by studies relying on limited sampling or single diagnostic markers. Multilocus phylogenetic analyses consistently resolved four well-supported and genetically distinct lineages within Clade B of *Pythium* species, designated *P. macrosporangium*, *P. melonis*, *P. tardioosporum*, and *P. tumidoantherum*, and these lineages were further evaluated through detailed morphological observations, ecological distribution patterns, and pathogenicity assays.

The nuclear ribosomal ITS region was not employed in this study because it has repeatedly been shown to lack sufficient resolution for species delimitation within *Pythium* Clade B [8,47,48], where closely related taxa often share identical or near-identical ITS sequences. Consistent with previous reports highlighting the limited resolution of the ITS region within specific lineages of *Pythium* Clade B, preliminary ITS sequencing in our study failed to separate *P. macrosporangium* from *P. afertile*, *P. melonis* and *P. tumidoantherum* from *P. contiguanum*, or *P. tardioosporum* from *P. rhizo-oryzae*. Reliance on ITS in this group can therefore obscure true phylogenetic diversity and lead to underestimation of species boundaries, necessitating the use of more informative mitochondrial and nuclear loci. Importantly, our results revealed marker-specific differences in phylogenetic resolution, highlighting the limitations of relying on a single barcode for species delimitation within this group. In particular, the *cox1* phylogeny was unable to successfully differentiate *P. melonis* and *P. tumidoantherum* from each other, despite the fact that both lineages are clearly distinct from *P. contiguanum* based on a combination of stable morphological traits and multilocus molecular evidence. In contrast, the *cox2* phylogeny provided clear and complete separation of *P. melonis* and *P. tumidoantherum* as independent lineages, with high bootstrap and posterior probability supports, underscoring the superior discriminatory power of *cox2* for resolving closely related taxa within Clade B [47].

Morphological evidence further supports the distinction of *P. melonis* and *P. tumidoantherum* from their closely related species, as *P. contiguanum* is characterized by contiguous, inflated, catenulate sporangia associated with hyphal elements and by active zoospore production [49], whereas neither of these diagnostic features was observed in *P. melonis* or *P. tumidoantherum*. Instead, *P. melonis* produces strictly filamentous sporangia consistent with the classical morphology of *Pythium sensu stricto*, while *P. tumidoantherum* does not form sporangia but produces only intercalary hyphal swellings; despite several attempts, no zoospore production was observed. In addition, the two lineages differ clearly in their sexual structures, including oogonial and antheridial characters, indicating stable reproductive divergence between *P. melonis* and *P. tumidoantherum* and further excluding conspecificity with *P. contiguanum*. An additional line of evidence distinguishing *P. melonis* and *P. tumidoantherum* from *P. contiguanum* lies in their contrasting ecological and physiological roles. *Pythium contiguanum* has been demonstrated to function as an antagonist with proven biocontrol activity rather than as a plant pathogen [49], whereas both *P. melonis* and *P. tumidoantherum* consistently induced disease symptoms on *C. melo* var. *reticulatus* in the present study. Thus, beyond stable morphological characters and clear separation in *cox2* phylogenies, these taxa differ fundamentally in their capacity to colonize hosts and cause disease.

A similar pattern of marker-specific resolution was observed for *P. macrosporangium*, which was not differentiated from *P. afertile* in *cox1*-based phylogenetic analyses, a result that could misleadingly suggest species identity if considered in isolation. However, *P. macrosporangium* readily and consistently produced sexual structures, whereas *P. afertile* is characterized by the absence of sexual reproduction, representing a fundamental biological and morphological distinction between the two taxa. Moreover, the *cox2* phylogeny completely and unambiguously separated *P. macrosporangium* from *P. afertile*, with strong statistical support, confirming that the lack of resolution in *cox1* reflects marker limitations rather than true taxonomic affinity. These findings collectively demonstrate that *cox1* alone is insufficient for reliable species delimitation within Clade B and reinforce the necessity of multilocus approaches for accurate taxonomic inference in *Pythium* [8,47].

In addition, this study documented notable intraspecific morphological plasticity within *P. tardioosporum*, particularly with respect to the shape and position of hyphal swellings, which varied among isolates and included globose, subglobose, and irregular forms; some isolates produced a mixture of these types, whereas others formed exclusively irregular swellings, and their position along the hyphae ranged from terminal to intercalary, or both. A similar degree of plasticity was observed in the antheridial configuration of *P. tardioosporum*, which was predominantly monoclinous across isolates; however, in isolate CP83, diclinous antheridia were also observed, indicating that antheridial origin is not invariant within this lineage. Morphological plasticity has been observed in other Clade B members, such as *P. plurisporium* Abad, Shew, Grand and L.T. Lucas and *P. banihashemianum* Mostowf. and Salmanin [48,50]. Despite this variability, *P. tardioosporum* formed a coherent and well-supported clade in multilocus phylogenetic analyses, indicating that the observed plasticity reflects intraspecific variation rather than taxonomic heterogeneity.

Among the four lineages, *P. macrosporangium* was particularly notable not only for its phylogenetic distinctness but also for its consistent production of globose to subglobose sporangia, a character that is exceptional within *Pythium sensu stricto*, where filamentous sporangia have long been regarded as diagnostic [5,6,7,47]. Although globose sporangia were also observed in *P. bostanabadense* Ahadi, A.C. Bouket, Alizadeh, Masigol and Grossart [51], *P. macrosporangium* is clearly distinguished from this species by its other morphological characteristics as well as its sequence data. The stable placement of *P. macrosporangium* within Clade B based on nuclear and mitochondrial loci indicates that sporangial morphology in *Pythium* spp. is more evolutionarily labile than traditionally assumed, and that reliance on classical morphological concepts alone may obscure significant phylogenetic and ecological diversity. Examples of this phenomenon were observed in *P. heteroogonium* Mostowf. and Salmanin., in which two distinct types of oogonia, smooth and ornamented, were present. Although approximately 70% of the oogonia in this species are smooth, it is classified within Clade D, whose members are characterized by exclusively ornamented oogonia [50].

Ecological differentiation among substrates strongly structured *Pythium* spp. communities, with pronounced contrasts in alpha diversity across irrigation water, rhizosphere soil, and root tissues. The extremely low diversity observed in irrigation water indicates strong environmental filtering, whereas the progressively higher diversity in soil and root tissues reflects increasing habitat complexity and host-mediated niche differentiation. Within this overall ecological framework, *P. macrosporangium* emerged as the dominant lineage across all sampled hosts, locations, and substrates. The exclusive recovery of *P. macrosporangium* from irrigation water highlights the potential of irrigation networks to serve as inoculum reservoirs and dispersal pathways for this pathogen. However, rather than reflecting strict environmental filtering, this pattern must be interpreted in light of the sample size and the inherent limits of isolation by baiting. It is noteworthy that previous studies showed that leaf baiting approach is less sensitive than metabarcoding in revealing the diversity of oomycete communities in environmental samples but effectively isolated the more actively sporulating and aggressive species [18,19]. Further studies combining larger aquatic surveys, water physicochemical analyses (e.g., pH and electrical conductivity), and environmental DNA (eDNA) metabarcoding are warranted to fully resolve oomycete community complexity and dynamics in irrigation systems. The occurrence of *P. macrosporangium* across all substrates and hosts, coupled with its unique persistence in irrigation water, highlights its high adaptability and long-term survival potential. These characteristics underscore the epidemiological importance of *P. macrosporangium* in greenhouse production systems, where irrigation water likely functions as a critical reservoir and inoculum spread pathway.

Although both symptomatic root and crown tissues were sampled and cultured during our initial field surveys, all primary isolates of these novel *Pythium* taxa were recovered exclusively from roots. Subsequent controlled pathogenicity assays, however, demonstrated their potential to colonize crown tissues under experimental inoculation conditions. This likely reflects the strong association of *Pythium* species with fine roots and the rhizosphere, where moisture, nutrients, and exposure to contaminated irrigation water favor infection and pathogen proliferation. Crown tissues may represent a less favorable or more transient niche, with lower pathogen biomass or reduced culturability. Pathogenicity assays showed that all four newly described *Pythium* species caused disease on *C. melo* var. *reticulatus*, producing pre-emergence damping-off and extensive root colonization. Their interactions with other cucurbit hosts were more variable and were often limited to low-level colonization without visible symptoms. *Pythium macrosporangium* showed the broadest pathogenic profile, causing pre-emergence damping-off on several hosts, whereas *P. melonis*, *P. tardioosporum*, and *P. tumidoantherum* were mostly restricted to asymptomatic colonization or failed establishment on non-melon hosts. These results suggest that pathogenicity in these Clade B species is host-dependent and mainly associated with root colonization, while crown colonization alone does not necessarily lead to disease. Similar patterns have been reported for other *Pythium* species, including *P. aphanidermatum* (Edson) Fitzp. from Clade A, indicating that cucurbit pathogenicity is not restricted to one phylogenetic lineage [15,52]. Boxplot and NMDS analyses further supported these patterns, showing species-specific differences in colonization and damping-off incidence. *Pythium macrosporangium* had higher median values and greater variability across traits, whereas *P. melonis* and *P. tardioosporum* showed low colonization frequencies and limited disease development. From an ecological standpoint, the multivariate trait patterns revealed by NMDS and PERMANOVA should be interpreted within the context of our analytical framework. Because isolates lacking measurable activity across all three disease parameters were excluded to prevent mathematical distortion in Bray–Curtis dissimilarity matrices, this ordination and permutational analyses specifically capture the trait syndromes and host-associated differentiation of actively pathogenic and colonizing isolates. Consequently, this subset-based approach does not encompass entirely non-pathogenic or strictly saprophytic members of the Clade B assemblage, bounding our ecological conclusions primarily to the virulence and colonization dynamics of competent isolates rather than total niche occupancy. Overall, the species × host interaction indicates that disease expression is context-dependent and shaped by host-specific compatibility.

## 5. Conclusions

Collectively, the results of this study reveal previously unrecognized taxonomic complexity within *Pythium* Clade B, confirm the necessity of multilocus phylogenetic frameworks for species delimitation, document significant yet structured morphological plasticity within lineages, and identify a morphologically exceptional and ecologically dominant lineage with major implications for oomycete systematics, diagnostics, and disease management in greenhouse agriculture. Future studies should expand geographic and host sampling and incorporate population-level genomic analyses to obtain a better insight into the distribution, genetic structure, and evolutionary dynamics of these newly described lineages.

## Figures and Tables

**Table 1:** List of the isolates used in this study with their GenBank accession numbers.

Species	Isolate	Date	Host (Matrix)	Location	Longitude	Latitude	GenBank Accession No.		
							*βtub*	*cox1*	*cox2*
*Pythium macrosporangium*									
	CP67	Oct. 2025	*Cucurbita pepo* (root tissue)	Beyza	29.99869	52.3808	PZ305329	PZ277325	PZ277342
	CP52	Oct. 2025	*Cucurbita pepo* (rhizosphere soil)	Arsanjan	29.96084	52.35114	PZ305330	PZ277324	PZ277341
	CP50	Dec. 2025	*Cucurbita pepo* (irrigation water)	Shiraz	29.81888	52.61608	PZ305331	PX985171	PZ277340
	CP102	Dec. 2025	*Cucurbita pepo* (irrigation water)	Shiraz	29.81808	52.61245	-	-	-
	CP104	Dec. 2025	*Cucurbita pepo* (irrigation water)	Marvdasht	29.88605	52.53937	-	-	-
	CP117	Dec. 2025	*Cucurbita pepo* (rhizosphere soil)	D. Arjan **	30.08008	51.52922	-	-	-
	CP202	Dec. 2025	*Cucurbita pepo* (rhizosphere soil)	Kazerun	30.0333	52.43562	-	-	-
	CP107	Dec. 2025	*Cucurbita pepo* (rhizosphere soil)	D. Arjan	30.07327	51.51064	-	-	-
	CP80	Dec. 2025	*Cucurbita pepo* (rhizosphere soil)	Neyriz	29.58851	52.84401	-	-	-
	CS112	Dec. 2025	*Cucumis sativus* (irrigation water)	Fasa	29.9042	52.54625	-	-	-
	CP474	Dec. 2025	*Cucurbita pepo* (irrigation water)	Kazerun	30.08008	51.52922	-	-	-
	CP476	Dec. 2025	*Cucurbita pepo* (irrigation water)	Neyriz	29.58851	52.84401	-	-	-
	CP477	Dec. 2025	*Cucurbita pepo* (irrigation water)	Arsanjan	29.9886	52.39963	-	-	-
	CP479	Dec. 2025	*Cucurbita pepo* (irrigation water)	Beyza	29.9042	52.54625	-	-	-
	CP481	Dec. 2025	*Cucurbita pepo* (irrigation water)	Arsanjan	29.96596	52.40623	-	-	-
	CP482	Dec. 2025	*Cucurbita pepo* (rhizosphere soil)	Sarvestan	29.90205	52.18789	-	-	-
	CP485	Dec. 2025	*Cucurbita pepo* (rhizosphere soil)	Beyza	30.00497	52.39841	-	-	-
	CP333	Dec. 2025	*Cucurbita pepo* (root tissue)	Shiraz	29.81525	52.61633	-	-	-
	CP349	Dec. 2025	*Cucurbita pepo* (rhizosphere soil)	Beyza	30.00497	52.39841	-	-	-
*Pythium melonis*									
	CM39	Oct. 2025	*Cucumis melo* * (root tissue)	Arsanjan	30.00246	52.32934	PZ305320	PX985154	PZ277331
	CM40	Oct. 2025	*Cucumis melo* (root tissue)	Arsanjan	29.9638	52.36212	PZ305321	PX985155	PZ277332
	CM80	Dec. 2025	*Cucumis melo* (root tissue)	Marvdasht	30.0331	52.43562	PZ305322	PX985156	PZ277333
	CM89	Oct. 2025	*Cucumis melo* (root tissue)	Shiraz	29.81808	52.61245	-	-	-
*Pythium tardioosporum*									
	CP61	Nov. 2025	*Cucurbita pepo* (rhizosphere soil)	Beyza	30.00575	52.40603	PZ305326	PX985160	PZ277337
	CP83	Dec. 2025	*Cucurbita pepo* (root tissue)	D. Arjan	30.07327	51.51064	PZ305328	PX985161	PZ277339
	CM223	Nov. 2025	*Cucumis melo* (root tissue)	Fasa	29.9042	52.54625	PZ305327	PX985162	PZ277338
*Pythium tumidoantherum*									
	CP20	Oct. 2025	*Cucurbita pepo* (root tissue)	Beyza	30.00058	52.40604	PZ305323	PX985157	PZ277334
	CP84	Dec. 2025	*Cucurbita pepo* (root tissue)	Neyriz	29.58851	52.84401	PZ305325	PX985158	PZ277336
	CP129	Dec. 2025	*Cucurbita pepo* (root tissue)	Shiraz	29.81525	52.61633	PZ305324	PX985159	PZ277335
	CP111	Oct. 2025	*Cucurbita pepo* (root tissue)	Marvdasht	30.0331	52.43562	-	-	-

* *C. melo* = *C. melo* var. *reticulatus*; ** D. Arjan = Dasht-e- Arjan.

**Table 2:** Morphological comparison of novel *Pythium* species in this study with their closely related taxa from Clade B.

Characteristic		*P.* *macrosporangium*	*P.* *afertile*	*P.* *melonis*	*P. tumidoantherum*	*P. contiguanum*	*P.* *tardioosporum*	*P.* *rhizo-oryzae*
Hyphal width (µm)		4.8	3.7	5	3.1	5	4.7	5–6
Colony morphology on								
	CMA	Rad.	Rad.	NR	Subm.	Rad.	Chr.	Rad.
	CA	Rad.	NR	NR	NR	Rad.	Ros.	Rad.
	HSA	Ros.	NR	NR	NR	Chr.	Ros.	Ros.
	MEA	Ros.	NR	NR	NR	Ros.	Pet.	Med.
	PDA	Median	NR	Subm.	Subm.	Ros.	Med.	Ros.
Daily growth rate on PCA (mm)		25.8	16	12	10	28.1	28	27.7
Cardinal temperatures (°C)								
	Minimum	5	NR	NR	NR	5	5	5
	Optimum	35	NR	NR	NR	30	25	30
	Maximum	37	NR	NR	NR	35	40	35
Zoospore production		+	+	NF	+	NF	+	+
Sporangia								
	Shape	Irreg., Sack., Obo., Obp., Elo.	Cont., Fila., Infla.	NF	Cont.	NF	Fila., Infla., Cont., Lob.	Stri. fila
	Location	Inter.	NA	NF	NA	NF	NA	NA
	Length (µm)	120	NA	NF	NA	NF	NA	NA
	Width (µm)	32	NA	NF	NA	NF	NA	NA
Hyphal swellings								
	Shape	Subglo., Glo., Sack.	NF	Glo., Cat.	NF	Subglo.	Glo., Subglo., Irreg.	Glo.
	Location	Int.	NF	Ter., Int.	NF	Int.	Int., Ter.	Int.
	Width (µm)	32	NF	NI	NF	9.9	35	20
Appressoria								
	Shape	Irreg., Sack.	NF	NF	Sph.; Sick.	NF	Fus.	NF
Sexual behavior		Hom.	Sterile	Hom.	Hom., Sterile	Hom.	Hom.	Hom.
Oogonia								
	Surface	S.	NA	S.	S.	S.	S.	S.
	Shape	Glo., Subglo.	NA	Glo., Sph.	Sph.	Glo., Subglo., Amorph.	Glo	Glo.
	Location	Ter., Int.	NA	Ter., Int.	Ter., Int.	Mostly Int.	Ter.	Ter.
	Length (µm)	12	NA	NF	NF	NF	NF	3
	Width (µm)	23.2	NA	19.7	10–24	17.2	39	30.5
Antheridia								
	Shape	Sack.	NA	NS	NR	Fus.	Cro.	Club., Sack.
	Attachment	Para., Amp.	NA	Para.	Para.	Para., rarely Hypo.	Para.	Para.
	Type	Dic.	NA	Mono.-Dic.	Mono.	Dic.	Mono.	Dic.-Mono.
	Width (µm)	19.7	NA	NF	NR	NA	11.2	13.2
	No. per oogonium	1–4	NA	NF	1–5	1–3	1	1–5
Oospores								
	Shape	Glo., Subglo.	NA	Glo.	Sph.	Glo., mostly Abor.	Glo.	Glo.
	Type	Aple.	NA	Aple.	Ple.	Ple.	Ple.	Ple.
	No. per oogonium	1–2	NA	1	1	1	1	1
	Width (µm)	23.5	NA	16.7	10–24	17.2	39	30.5
	Wall (µm)	2.1	NA	1–2	1–2.5	1.5	2.9	3.3

Abor. = Aborted; Amorph. = Amorphous; Amp. = Amphigynous; Aple. = Aplerotic; Cat. = Catenulate; Chr. = Chrysanthemum; Conti. = Contiguous; Cro. = Crook-necked; Dic. = Diclinous; Elo. = Elongated; Fila. = Filamentous; Fus. = Fusiform; Glo. = Globose; Hom. = Homothallic; Hypo. = Hypogynous; Infla. = Inflated; Int. = Intercalary; Irreg. = Irregular; Lob. = Lobate; Med. = Median; Mono. = Monoclinous; NA = Not applicable; NF = Not formed; NR = Not reported; NS. = No specific shape; Para. = Paragynous; Pet. = Petaloid; Ple. = Plerotic; Rad. = Radial; Ros. = Rosette; S. = Smooth; Sack. = Sack-shaped; Sick. = Sickle-shaped; Sph. = Spherical; Str. fila. = Strictly filamentous; Subglo. = Subglobose; Subm. = Submerged; Ter. = Terminal.

## Data Availability

The datasets generated and/or analyzed during the current study are available in the Zenodo repository, [www.zenodo.org; 10.5281/zenodo.19588427]. All sequenced data are deposited in GenBank and their accession numbers can be observed within Table 1.

## Supplementary Materials

The following supporting information can be downloaded at https://www.mdpi.com/article/10.3390/jof12100748/s1, Figure S1: Phylogenetic relationships of novel *Pythium* species from cucurbits and 55 previously described *Pythium* spp. based on Bayesian analyses of the alignment of beta tubulin gene (*βtub*) sequences. Numbers above the branches represent posterior probabilities and bootstrap support based of the Maximum Likelihood and Minimum Evolution analyses, in the respective order; Figure S2: Phylogenetic relationships of *Pythium* species from cucurbits and 69 previously described *Pythium* spp. based on Bayesian analyses of the alignment of cytochrome c oxidase subunit I (*cox1*) sequences. Numbers above the branches represent posterior probabilities and bootstrap support based of the Maximum Likelihood and Minimum Evolution analyses, in the respective order; Figure S3: Phylogenetic relationships of *Pythium* species from cucurbits and 50 previously described *Pythium* spp. based on Bayesian analyses of the alignment of cytochrome c oxidase subunit II (*cox2*) sequences. Numbers above the branches represent posterior probabilities and bootstrap support based of the Maximum Likelihood and Minimum Evolution analyses, in the respective order; Table S1: List of primers with their PCR conditions used in this study [53,54]; Table S2: *Pythium sensu stricto* isolate codes and their GenBank accession numbers for phylogenetic analyses comparison; Table S3: Morphological characteristics of the novel *Pythium* species in this study; Table S4: Base pair (bp) differences across *βtub*, *cox1*, and *cox2* sequences showing the interspecific variation of *Pythium macrosporangium* [MAC] with its related species (i.e., *P. afertile* [AFE] and *P. kashmirense* [KAS]), *P. melonis* [MEL] with its related species (i.e., *P. contiguanum* [CON] and *P. tumidoantherum* [TUM]), *P. tardioosporum* [TAR] with its related species (i.e., *P. rhizo-oryzae* [RHI] and *P. catenulatum* [CAT]), and *P. tumidoantherum* [TUM] with its related species (i.e., *P. contiguanum* [CON]); Table S5: Two-way ANOVA for average growth rate of four *Pythium* species on PCA in nine different temperatures; Table S6: Permutational multivariate analysis of variance (PERMANOVA) based on Bray–Curtis dissimilarities, testing the effects of species, host, and their interaction on pathogenicity trait composition; Table S7: The results of one-way analysis of variance for eight pathogenicity traits and growth indices of *Cucumis melo* plants inoculated with different new *Pythium* species obtained from greenhouse cucurbits of Fars Province.

## Abbreviations

The following abbreviations are used in this manuscript:

ANOVA	Analysis of Variance
*βtub*	Beta tubulin
*C.*	*Cucumis*
CA	Carrot Agar
CMA	Corn Meal Agar
*Ci.*	*Citrullus*
*cox*	Cytochrome c oxidase
*Cu.*	*Cucurbita*
HSA	Hemp Seed Agar
ITS	Internal transcribed spacer of rDNA
MEA	Malt Extract Agar
NMDS	Non-metric Multidimensional Scaling
*P.*	*Pythium*
PCA	Potato Carrot Agar
PCNB	pentachloronitrobenzene
PDA	Potato Dextrose Agar
PERMANOVA	Permutational Multivariate Analysis of Variance
