# Supplementary material for: Taxonomy and Ecology of Novel Pythium sensu stricto Clade B Species Isolated from Cucurbits
Source: J Fungi (Basel). 2026 Oct 5;12(10):748. doi: 10.3390/jof12100748 (PMC13706351; doi:10.3390/jof12100748)
Supplement: Supplementary file 1 [file jof-12-00748-s001.zip › jof-4545826-supplementary.pdf]

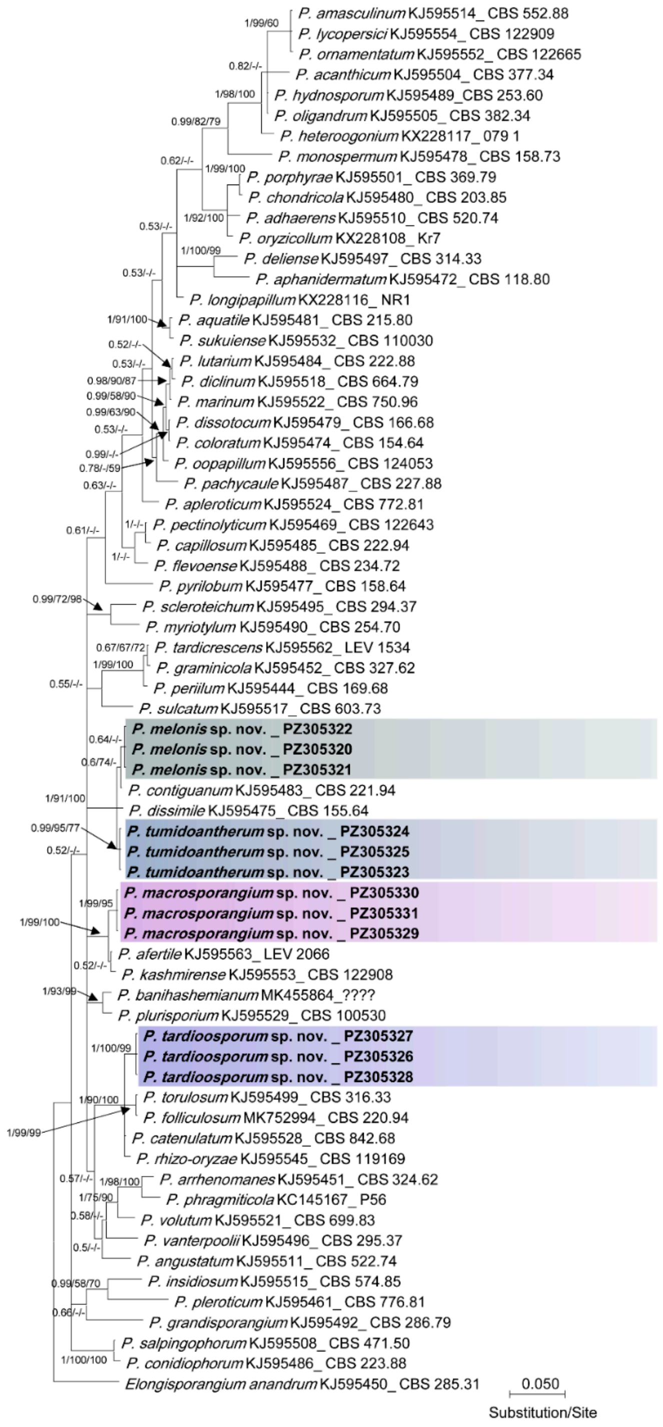

Figure S1. Phylogenetic relationships of novel *Pythium* species from cucurbits and 55 previously described *Pythium* spp. based on Bayesian analyses of the alignment of beta tubulin gene (*βtub*) sequences. Numbers above the branches represent posterior probabilities and bootstrap support based of the Maximum Likelihood and Minimum Evolution analyses, in the respective order.

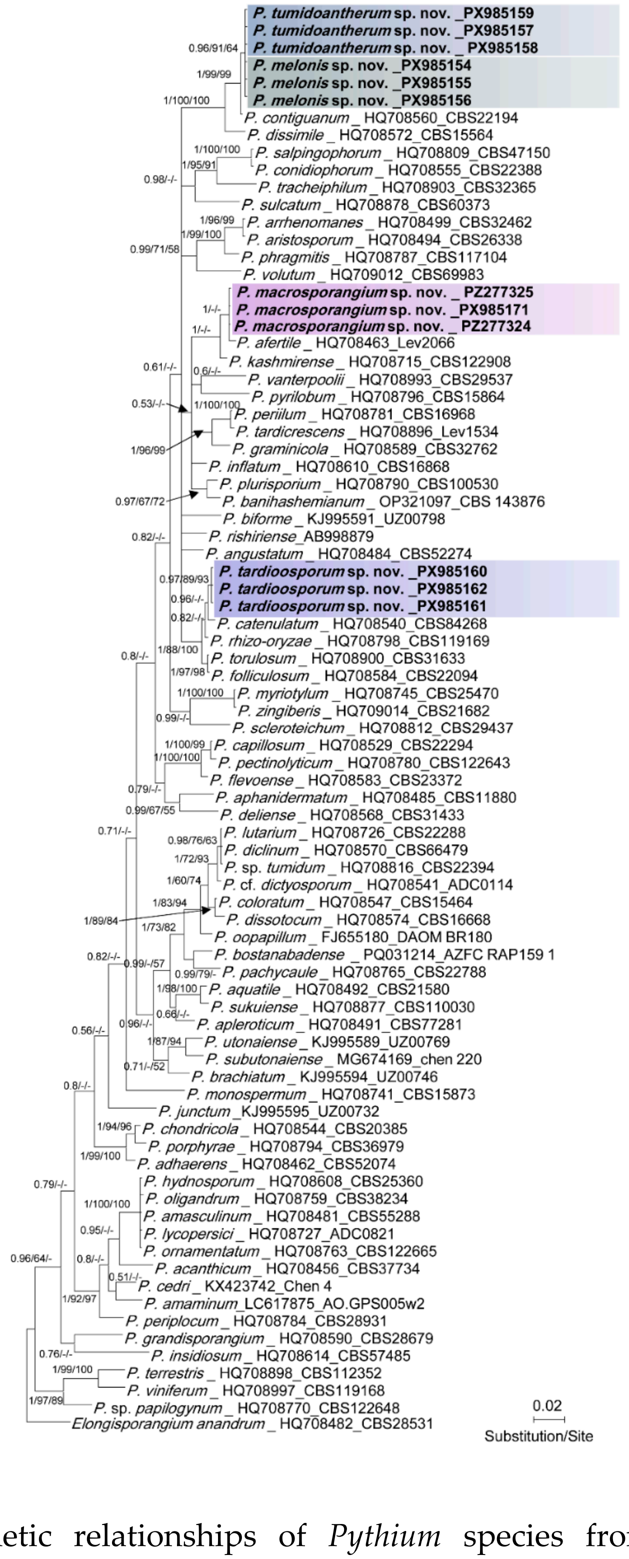

Figure S2. Phylogenetic relationships of *Pythium* species from cucurbits and 69 previously described *Pythium* spp. based on Bayesian analyses of the alignment of cytochrome c oxidase subunit I (*cox1*) sequences. Numbers above the branches represent posterior probabilities and bootstrap support based of the Maximum Likelihood and Minimum Evolution analyses, in the respective order.

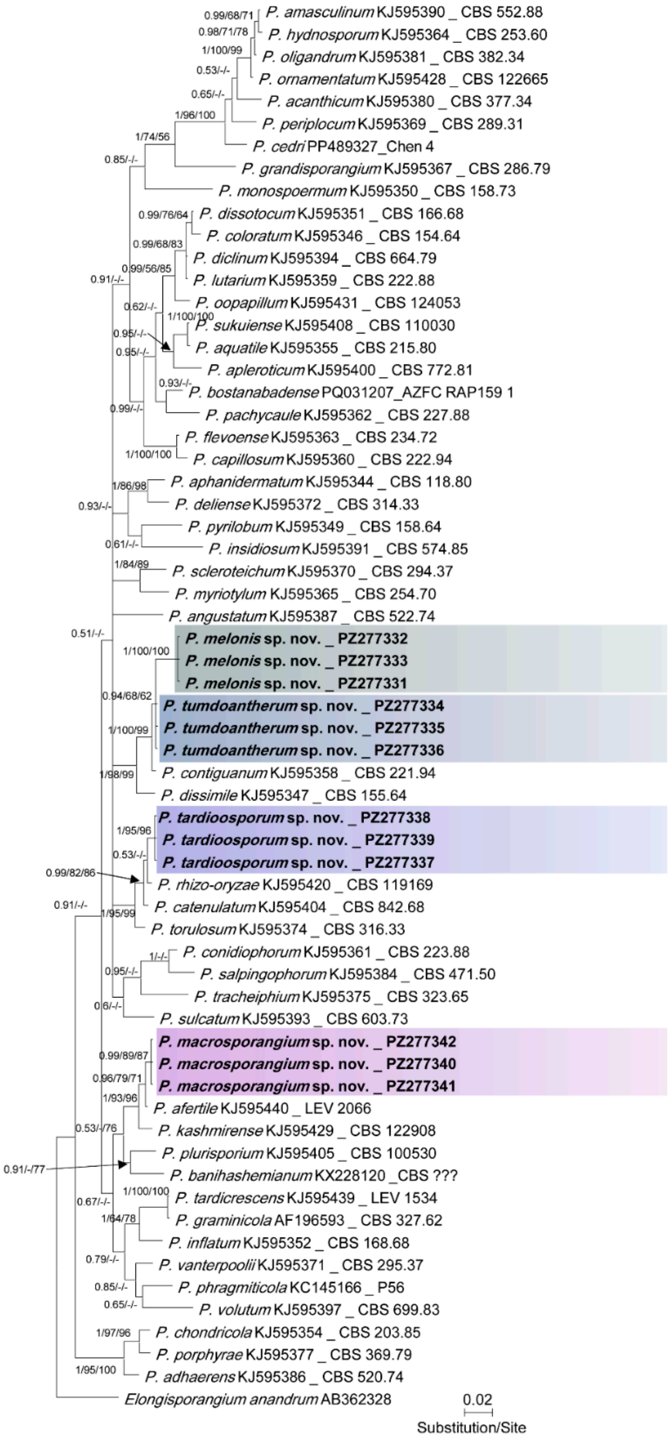

Figure S3. Phylogenetic relationships of *Pythium* species from cucurbits and 50 previously described *Pythium* spp. based on Bayesian analyses of the alignment of cytochrome c oxidase subunit II (*cox2*) sequences. Numbers above the branches represent posterior probabilities and bootstrap support based of the Maximum Likelihood and Minimum Evolution analyses, in the respective order.

Table S1. List of primers with their PCR conditions used in this study.

Target DNA	Primer name	Primer sequence (5'→3')	Reference	Initial denaturation	Number of cycles	Denaturation	Annealing	Extension	Final extension
βtub[a]	BT6<br>BT5	GTATCATGTGCACGTACTCGG<br>CAAGAAAGCCTTACGACGGA	Villa et al. 2006	95 (600)	30	95 (20)	63 (40)	72 (60)	72 (600)
cox1[b]	Oomcox1_Levup<br>Oomcox1_Levlo	TCAWCWMGATGGCTTTTTTCAAC<br>CYTCHGGRTGWCCRAAAAACCAAA	Robideau *et al.* 2011	95 (600)	30	95 (30)	51 (40)	72 (60)	72 (600)
cox2[c]	Cox2-F<br>Cox2-R	GGCAAATGGGTTTTCAAGATCC<br>CCATGATTAATACCACAAATTTCACTAC	Hudspeth *et al.* 2000	95 (600)	30	95 (30)	56 (30)	72 (60)	72 (600)

[a] β-tubulin. [b] cytochrome c oxidase subunit I, [c] cytochrome c oxidase subunit II, [e] temperature, °C (time, s).

Table S2. *Pythium sensu stricto* isolate codes and their GenBank accession numbers for phylogenetic analyses comparison.

Species	Isolate Code	GenBank Accession Numbers.		
		βtub	***cox1***	***cox2***
Elongisporangium anandrum	CBS 285.31	KJ595450	HQ708483	AB362328
P. acanthicum	CBS 377.34	KJ595504	HQ708458	KJ595380
P. adhaerens	CBS 520.74	KJ595510	HQ708462	KJ595386
P. afertile	LEV 2066	KJ595563	HQ708463	KJ595440
P. amaminum	AO.GPS005w2	-	LC617875	-
P. amasculinum	CBS 552.88	KJ595514	HQ708481	KJ595390
P. angustatum	CBS 522.74	KJ595511	HQ708484	KJ595387
P. aphanidermatum	CBS 118.80	KJ595472	HQ708486	KJ595344
P. apiculatum	CBS 120945	-	-	KJ595422
P. apleroticum	CBS 772.81	KJ595524	HQ708491	KJ595400
P. aquatile	CBS 215.80	KJ595481	HQ708493	KJ595355
P. aristosporum	ATCC 11101	AY598627	HQ708495	AB095060
P. arrhenomanes	CBS 324.62	KJ595451	HQ708519	AF196587
P. banihashemianum	CBS 143876	MK455864	OP321097	KX228120
P. biforme	UZ00798	-	KJ995591	-
P. bostanabadense	AZFC-RAP159-1	-	PQ031214	PQ031207
P. brachiatum	UZ00746	-	KJ995594	-
P. capillosum	CBS 222.94	KJ595485	HQ708529	KJ595360
P. catenulatum	CBS 842.68	KJ595528	HQ708536	KJ595404
P. cedri	Chen 4	-	KX423742	PP489327
P. cf. *dictyosporum*	ADC0114	-	HQ708541	-
P. chondricola	CBS 203.85	KJ595480	HQ708543	KJ595354
P. coloratum	CBS 154.64	KJ595474	HQ708553	KJ595346
P. conidiophorum	CBS 223.88	KJ595486	HQ708559	KJ595361
P. contiguanum	CBS 221.94	KJ595483	HQ708560	KJ595358
P. deliense	CBS 314.33	KJ595497	HQ708566	KJ595372
P. diclinum	CBS 664.79	KJ595518	HQ708569	KJ595394
P. dissimile	CBS 155.64	KJ595475	HQ708573	KJ595347
P. dissotocum	CBS 166.68	KJ595479	HQ708576	KJ595351
P. flevoense	CBS 234.72	KJ595488	HQ708583	KJ595363
P. folliculosum	CBS 22094	MK752994	HQ708584	-
P. graminicola	CBS 327.62	KJ595452	HQ708589	AF196593
P. grandisporangium	CBS 286.79	KJ595492	HQ708590	KJ595367
P. helicandrum	CBS 393.54	AY598653	-	AB362329
P. heteroogonium	CBS 141232	KX228117	-	KX228131
P. hydnosporum	CBS 253.60	KJ595489	HQ708608	KJ595364
P. inflatum	CBS 16868	AY598626	HQ708610	KJ595352
P. insidiosum	CBS 574.85	KJ595515	HQ708613	KJ595391

P. junctum	UZ00732	-	KJ995595	-
P. kashmirense	CBS 122908	KJ595553	HQ708715	KJ595429
P. longipapillum	CBS 141231	KX228116	-	KX228128
P. lutarium	CBS 222.88	KJ595484	HQ708726	KJ595359
P. lycopersici	CBS 122909	KJ595554	HQ708727	KJ595343
P. macrosporum	CBS 574.80	AY598646	HQ708730	AB512916
P. marinum	CBS 750.96	KJ595522	-	KJ595398
P. marsipium	CBS 773.81	AY598699	HQ708734	KJ595401
P. monospermum	CBS 158.73	KJ595478	HQ708743	KJ595350
P. myriotylum	CBS 254.70	KJ595490	HQ708748	KJ595365
P. oligandrum	CBS 382.34	KJ595505	HQ708760	KJ595381
P. oopapillum	CBS 124053	KJ595556	FJ655180	KJ595431
P. ornamentatum	CBS 122665	KJ595552	HQ708763	KJ595428
P. oryzicollum	CBS 140941	KX228108	-	KX228125
P. pachycaule	CBS 227.88	KJ595487	HQ708767	KJ595362
P. pectinolyticum	CBS 122643	KJ595469	HQ708780	-
P. periilum	CBS 16968	KJ595444	HQ708781	-
P. phragmiticola	P56	KC145167	-	KC145166
P. phragmitis	CBS 117104	-	HQ708787	AJ890351
P. plurisporium	CBS 100530	KJ595529	HQ708790	KJ595405
P. porphyrae	CBS 369.79	KJ595501	HQ708794	KJ595377
P. prolatum	CBS 845.68	AY598652	-	AB362330
P. pyrilobum	CBS 158.64	KJ595477	HQ708796	KJ595349
P. recalcitrans	CBS 122440	-	-	KJ595423
P. rhizo-oryzae	CBS 119169	KJ595545	HQ708798	KJ595420
P. salpingophorum	CBS 471.50	KJ595508	HQ708809	KJ595384
P. schmitthenneri	CBS 129726	-	-	JF895530
P. scleroteichum	CBS 294.37	KJ595495	HQ708812	KJ595370
P. senticosum	CBS 122490	HQ643773	-	AB362317
P. sp. *spiculacarpum*	CBS 122647	-	HQ708815	-
P. sp. *tumidum*	CBS 223.94	EF583440	HQ708816	-
P. subutonaiense	Chen 220a	-	MG674169	-
P. sukuiense	CBS 110030	KJ595532	HQ708877	KJ595408
P. sulcatum	CBS 603.73	KJ595517	HQ708878	KJ595393
P. tardicrescens	LEV 1534	KJ595562	HQ708896	KJ595439
P. torulosum	CBS 316.33	KJ595499	HQ708900	KJ595374
P. tracheiphilum	CBS 32365	-	HQ708903	KJ595375
P. utonaiense	UZ00758	-	KJ995588	-
P. vanterpoolii	CBS 295.37	KJ595496	HQ708991	KJ595371
P. volutum	CBS 699.83	KJ595521	HQ709012	KJ595397
P. zingiberis	CBS 21782	-	HQ709013	-

- = no data available.

Table S3. Morphological characteristics of the novel *Pythium* species in this study.

**Characteristic**	***P. macrosporangium***			***P. melonis***			***P. tardioosporum***			***P. tumidoantherum***		
	**CP50**	**CP22**	**CP25**	**CM39**	**CM40**	**CM80**	**CP61**	**CP83**	**CM223**	**CP20**	**CP84**	**CP129**
Hyphal width (µm)	4.8	3	6.5	5	5.4	4.6	4.7	5.1	4	3.1	2.2	3
Colony morphology on												
CMA	Rad.	Rad.	Rad.	Rad.	Rad.	Chr.	Chr.	Chr.	Chr.	Rad.	Rad.	Rad.
CA	Rad.	Rad.	Rad.	Rad.	Rad.	Ros.	Ros.	Ros.	Ros.	Rad.	Rad.	Rad.
HSA	Ros.	Ros.	Ros.	Ros.	Ros.	Ros.	Ros.	Ros.	Ros.	Chr.	Chr.	Chr.
MEA	Ros.	Ros.	Ros.	Med.	Med.	Pet.	Pet.	Pet.	Pet.	Ros.	Ros.	Ros.
PDA	Med.	Med.	Med.	Ros.	Ros.	Med.	Med.	Ros.	Ros.	Ros.	Ros.	Ros.
Daily growth rate on PCA (mm)	25.8	26	25.5	27.7	27	27.5	28	27.5	27.4	27.4	27.9	28
Cardinal temperatures (°C)												
Minimum	5	5	5	5	5	5	5	5	5	5	5	5
Optimum	35	35	35	30	30	25	25	25	25	30	30	30
Maximum	37	37	37	35	35	40	40	40	40	35	35	35
Zoospore production	+	+	+	+	+	+	+	+	+	+	NF	NF
Sporangia												
Shape	Irreg., Sack., Obo., Obp., Elo.	Irreg., Sack., Obo., Obp., Elo.	Irreg., Sack., Obo., Obp., Elo.	Str. fila.	Stri. fila.	Fila., Infla., Cont., Lob.	Fila., Infla., Cont., Lob.	Fila., Infla., Cont., Lob.	Fila., Infla., Cont., Lob.	NF	NF	NF
Location	Int.	Int.	Int.	NA	NA	NA	NA	NA	NA	NF	NF	NF
Length (µm)	120	160	99	NA	NA	NA	NA	NA	NA	NF	NF	NF
Width (µm)	32	72	41	NA	NA	NA	NA	NA	NA	NF	NF	NF
Hyphal swellings												
Shape	Subglo., Glo., Sack,	Subglo., Glo., Sack,	Subglo., Glo., Sack,	Glo.	Subglo.	Glo., Subglo., Irreg.	Glo., Subglo., Irreg.	Glo., Irreg.	Irreg.	Subglo.	Subglo.	Subglo.
Location	Int.	Int.	Int.	Int.	Int.	Int., Ter.	Int., Ter.	Ter., Int.	Int.	Int.	Int.	Int.
Width (µm)	13.5	22.7	30	20	21	35	35	34.1	36	9.9	10.8	8.9
Appressoria												
Shape	Irreg., Sack.	Irreg., Sack.	Irreg., Sack.	NA	NA	Fus.	Fus.	Fus.	Fus.	NA	NA	NA
Sexual behavior	Hom.	Hom.	Hom.	Hom.	Hom.	Hom.	Hom.	Hom.	Hom.	Hom.	Hom.	Hom.
Oogonia												
Surface	S. Glo, Subglo.	S. Glo., Subglo.	S. Glo., Subglo.	S. Glo.	S. Glo.	S. Glo.	S. Glo.	S. Glo.	S. Glo.	S. Glo., Subglo., Amorph.	S. Glo., Subglo., Amorph.	S. Glo., Subglo., Amorph.
Shape	Ter., Int.	Ter., inter	Ter., Int.	Ter.	Ter., Chain.	Ter.	Ter.	Ter.	Ter.	Mostly Int.	Int.	Int.
Location	12	17	19.3	3	NF	NF	NF	NF	NF	NF	NF	NF
Papilla (µm)	23.2	27.1	24.8	30.5	30	39	39	37	41	17.2	16.7	20.5
Antheridia												
Shape	Sack.	Sack.	Sack.	Club., Sack.	Club.	Cro.	Cro.	Cro.	Cro.	Fusi.	Fus.	Fus.
Attachment	Para., Amp.	Para., Amp.	Para., Amp.	Para.	Para.	Para.	Para.	Para.	Para.	Para., rarely Hypo.	Para., rarely Hypo.	Para., rarely Hypo.
Type	Dic.	Dic.	Dic.	Dic.-Mono.	Dic.	Mono.	Mono.	Mono.-Dic.	Mono.	Dic.	Dic.	Dic.

Width (μm)	19.7	19.7	19.7	13.2	10.1	11.2	11.2	7.8	14.7	NA	NA	NA
No. per oogonium	1–4	1–4	1–4	1–5	1–5	1	1	1	1	1–3	1–3	1–3
Oospores												
Shape	Glo., Subglo.	Glo., Subglo.	Glo., Subglo.	Glo.	Glo.	Glo.	Glo.	Glo.	Glo.	Glo., mostly Abor.	Glo., mostly Abor.	Glo., mostly Abor.
Type	Aple.	Aple.	Aple.	Ple.	Ple.	Ple.	Ple.	Ple.	Ple.	Ple.	Ple.	Ple.
No. per oogonium	1–2	1–2	1–2	1	1	1	1	1	1	1	1	1
Width (μm)	23.5	20.1	43.1	30.5	29.4	39	39	37	41	17.2	16.7	20.5
Wall (μm)	2.1	2.5	1.3	3.3	2.8	2.9	2.9	3.5	2.2	1.5	3	0.9

Abor. = Aborted; Amorph. = Amorphous; Amp. = Amphigynous; Aple. = Aplerotic; Chain. = formed in Chains; Chr. = Chrysanthemum; Conti. = Contiguous; Cro. = Crook-necked; Dic. = Diclinous; Elo. = Elongated; Fila = Filamentous; Fus. = Fusiform; Glo. = Globose; Hom. = Homothallic; Hypo. = Hypogynous; Infla. = Inflated; Int. = Intercalary; Irreg. = Irregular; Lob. = Lobate; Med. = Median; Mono. = Monoclinous; NA = Not applicable; NF = Not formed; Para. = Paragynous; Pet. = Petaloid; Ple. = Plerotic; Rad. = Radial; Ros. = Rosette; S. = Smooth; Sack. = Sack-shaped; Sph. = Spherical; Str. fila. = Strictly filamentous; Subglo. = Subglobose; Ter. = Terminal.

Table S4. Base pair (bp) differences across *βtub*, *cox1*, and *cox2* sequences showing the interspecific variation of *Pythium macrosporangium* [MAC] with its related species (i.e., *P. afertile* [AFE] and *P. kashmirense* [KAS]), *P. melonis* [MEL] with its related species (i.e., *P. contiguanum* [CON] and *P. tumidoantherum* [TUM]), *P. tardioosporum* [TAR] with its related species (i.e., *P. rhizo-oryzae* [RHI] and *P. catenulatum* [CAT]), and *P. tumidoantherum* [TUM] with its related species (i.e., *P. contiguanum* [CON]).

Region	Alignment length (bp)	Differences MAC/AFE (bp)	Differences MAC/KAS (bp)	Differences MEL/CON (bp)	Differences MEL/TUM (bp)	Differences TAR/CAT (bp)	Differences TAR/RHI (bp)	Differences TUM/CON (bp)	Differences within MAC (bp)	Differences within MEL (bp)	Differences within TUM (bp)	Differences within TAR (bp)
cox1	660	3	83	1	0	5	4	2	0	0	0	0
cox2	532	4	11	40	10	4	4	50	0	0	0	0
βtub	564	5	6	8	1	15	15	5	0	0	0	0
Total	1756	12	100	49	11	24	23	57	0	0	0	0

Table S5. Two-way ANOVA for average growth rate of four *Pythium* species on PCA in nine different temperatures.

SOV	df	Average growth rate		
		**MS**	**F-value**	***p***
Species	3	9.397551	496.781517711325	<0.0001
Temperature	8	69.96693	3698.65279726448	<0.0001
Species × Temperature	24	8.411329	444.646985994032	<0.0001
Residuals	72	0.018917		
Coefficient of variation (%)			2.64	

df: degrees of freedom; F: Fischer's statistics; MS: mean square; *p*: probabilities tested using Fisher's exact test; SOV: source of variation.

Table S6. Permutational multivariate analysis of variance (PERMANOVA) based on Bray–Curtis dissimilarities, testing the effects of species, host, and their interaction on pathogenicity trait composition.

**Source**	**df**	**Sum of squares**	**R^2**	**F-value**	***p***
Species	3	0.8488	0.162	9.70	0.001 ***
Host	4	3.9288	0.749	33.69	0.001 ***
Species × Host	6	0.8413	0.160	26.16	0.001 ***
Residuals	28	0.1501	0.029	—	—
Total	41	5.2479	1.000	—	—

df = degrees of freedom; F: Fischer’s statistics; *p*: probabilities tested using Fisher’s exact test; R^2 = proportion of variation explained by each factor.

Table S7. The results of one-way analysis of variance for eight pathogenicity traits and growth indices of *Cucumis melo* plants inoculated with different new *Pythium* species obtained from greenhouse cucurbits of Fars Province.

SOV	df	Root wet weight (g)			Foliage wet weight (g)			Root dry weight (g)		
		MS	F-value	*p*-value	MS	F-value	*p*	MS	F-value	*p*
Treatment	4	0.49	18.11	<0.0001	0.93	40.79	<0.0001	0.49	26.05	<0.0001
Error	10	0.028			0.023			0.019		
**CV (%)**			**41.21**			**25.93**			**38.66**	

Table S7. Continued.

SOV	df	Foliage dry weight (g)			Root length (mm)			Plant height (mm)		
		MS	F-value	*p*	MS	F-value	*p*	MS	F-value	*p*
Treatment	4	0.88	35.31	<0.0001	118.23	11.98	0.0008	426.57	10.65	0.0013
Error	10	0.025			9.9			40.07		
**CV (%)**			**29.97**			**20.4**			**10.22**	

Table S7. Continued.

SOV	df	Biomass wet weight (g)			Biomass dry weight (g)		
		MS	F-value	*p*	MS	F-value	*p*
Treatment	4	1.37	35.77	<0.0001	1.33	38.14	<0.0001
Error	10	0.038			0.035		
**CV (%)**			**27.01**			**28.81**	

CV: Coefficient of variation; df: Degrees of freedom; MS: Mean Square; SOV: Source of variation.
